# Supplementary figures and images for: EXP1 is critical for nutrient uptake across the parasitophorous vacuole membrane of malaria parasites
Source: PLoS Biol. 2019 Sep 30;17(9):e3000473. doi: 10.1371/journal.pbio.3000473 (PMC6786648; doi:10.1371/journal.pbio.3000473)

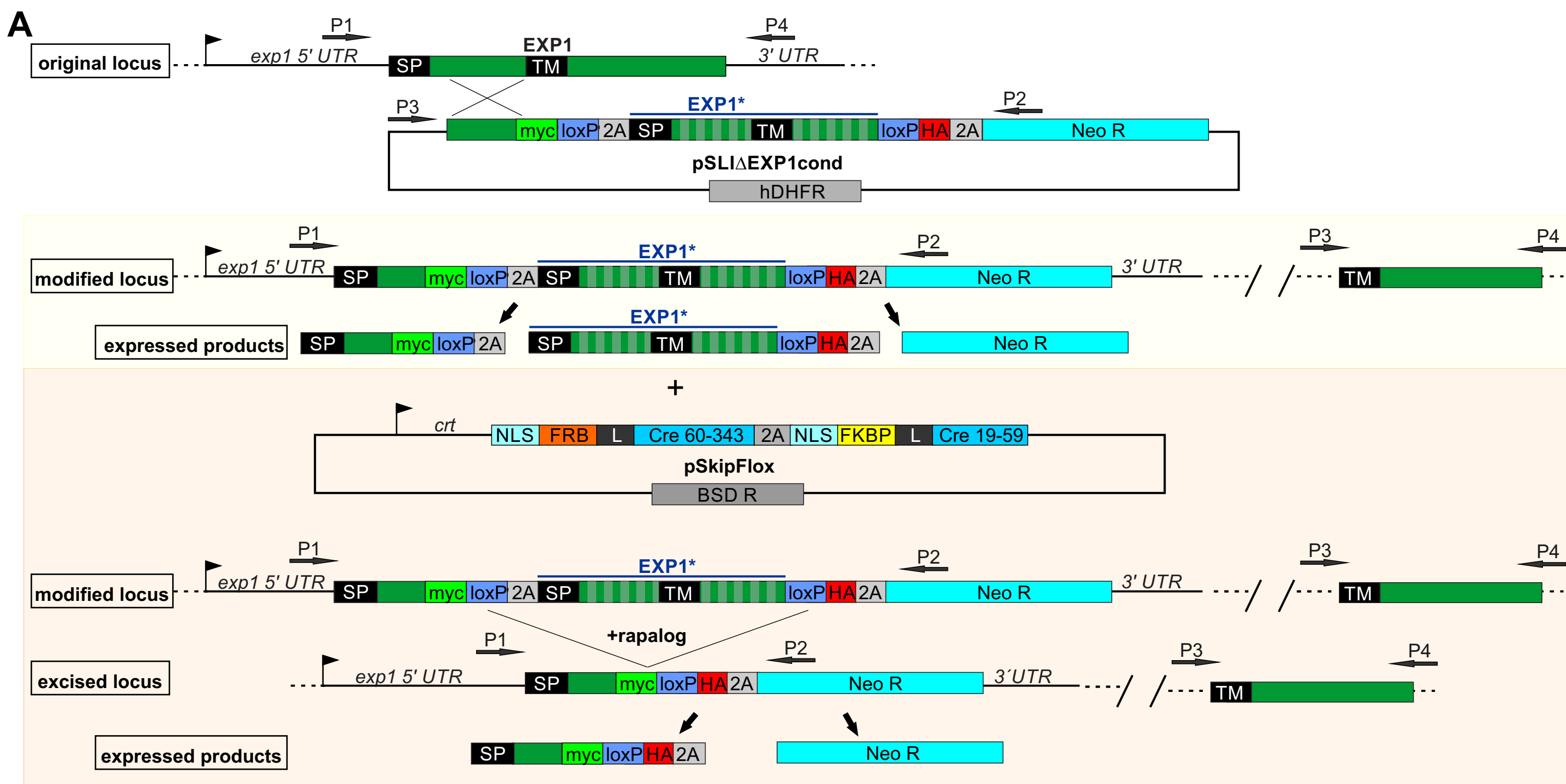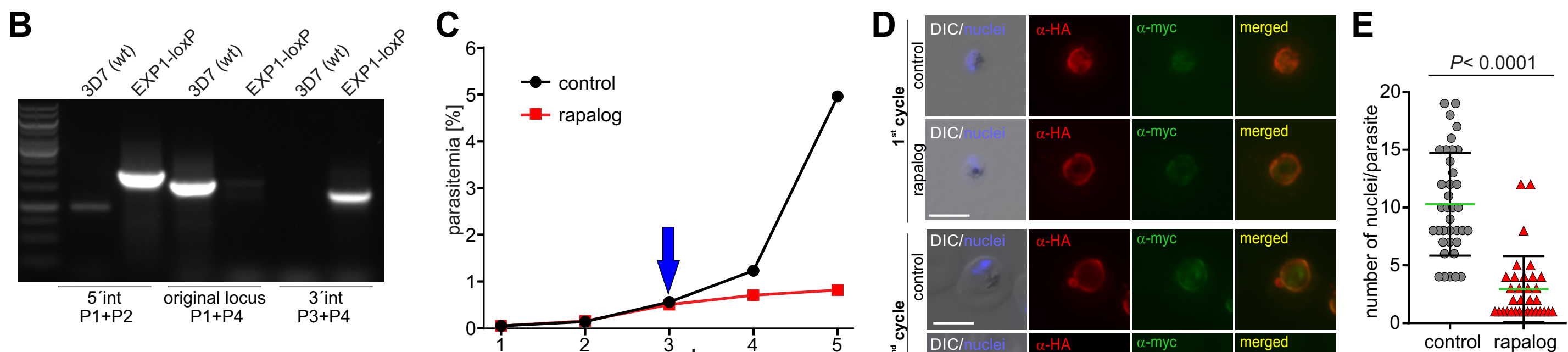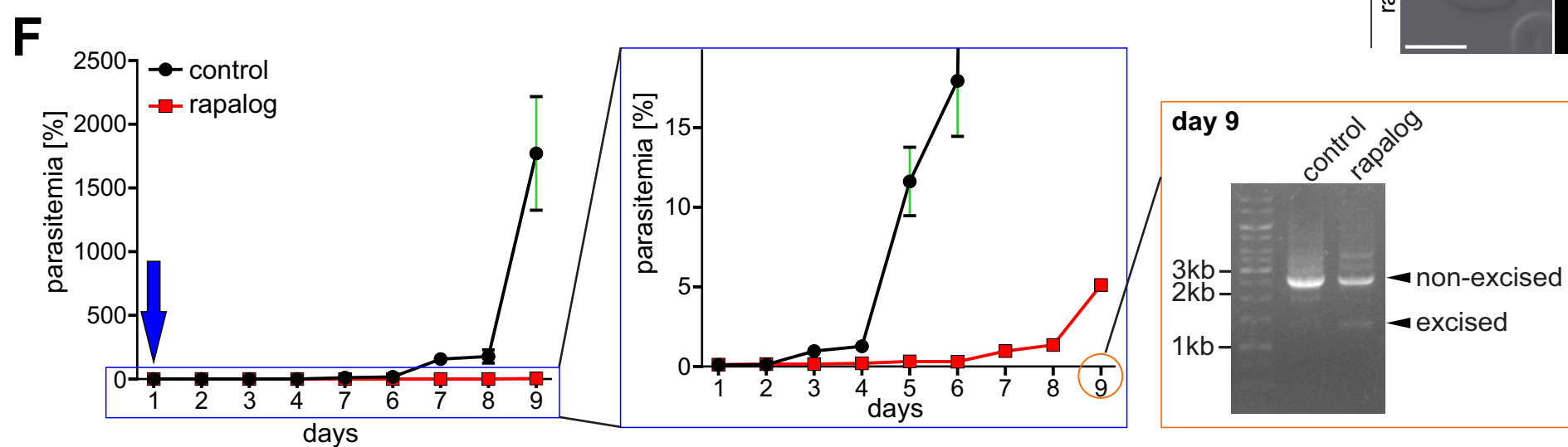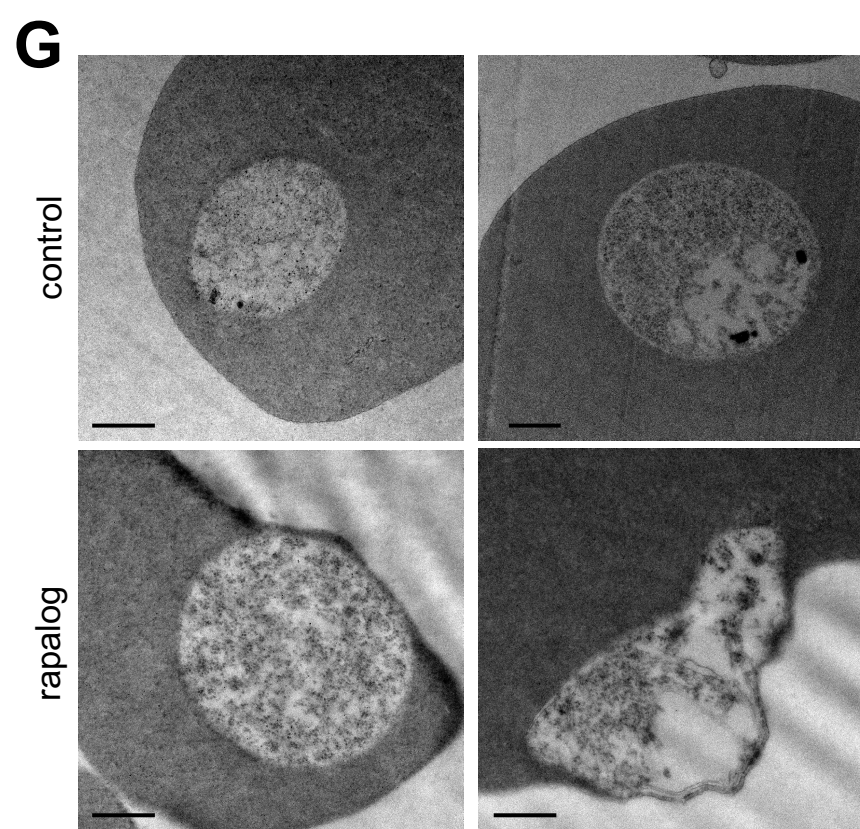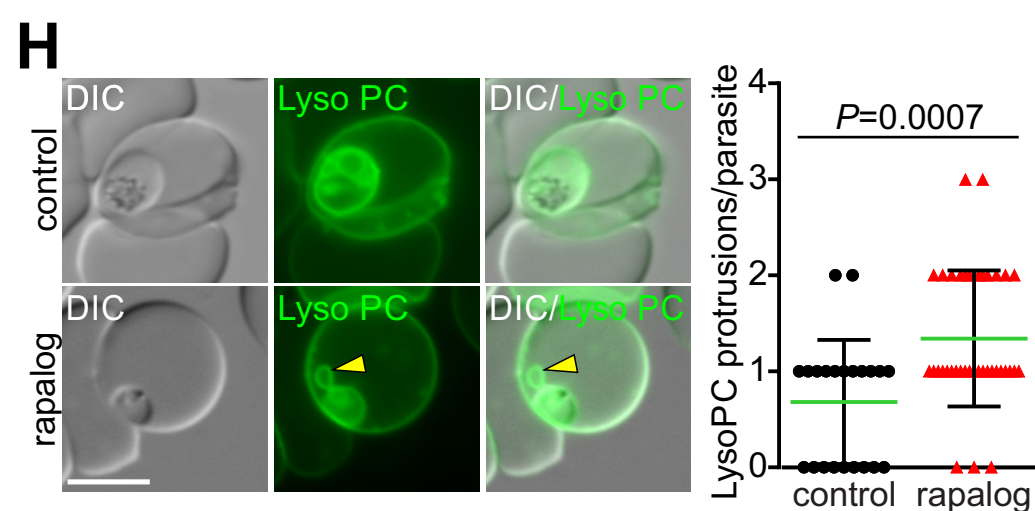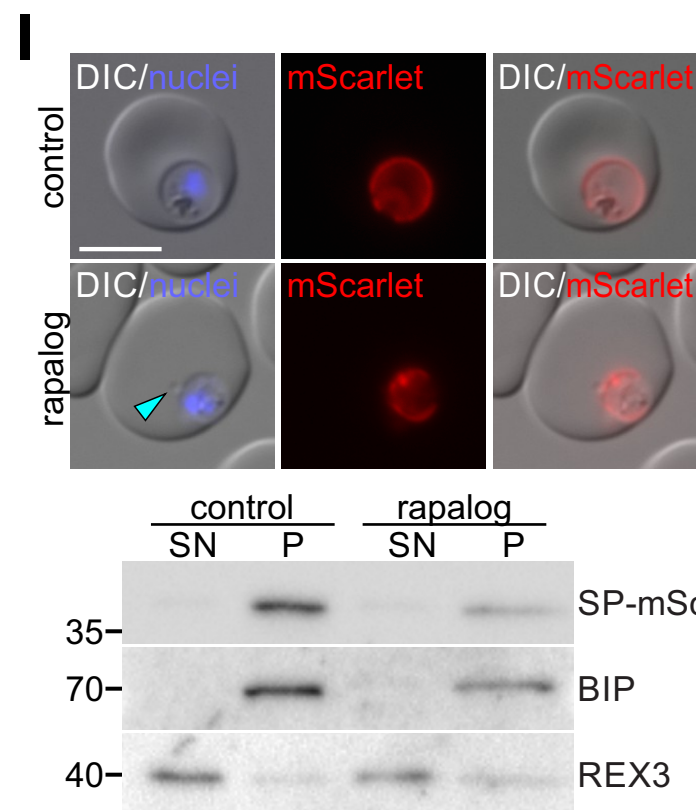

Supplement: S1 Fig — (A) Schematic representation of the SLI strategy to obtain a cell line for DiCre-based conditional KO of exp1. Top shows endogenous locus and plasmid pSLIΔEXP1cond. Light yellow box: cell line after SLI. Light orange box: the cell line transfected with pSkipFlox [20] and after induction of DiCre. “loxP” indicates the loxP site; asterisks indicates in-frame stop codon. Cre 60–343, Cre 19–59: Cre fragments; arrows, primers P1, P2, P3, and P4. (B) PCR on gDNA of condΔEXP1 and 3D7 parasites using the primers indicated in (a) confirming: 5'Int, 5'integration; absence of 'original locus'; 3'Int, 3' integration. (C) FC growth curves of synchronous condΔEXP1 ring stage parasites grown ± rapalog over 5 days (addition of rapalog starting day 1). Blue arrow indicates start of cycle without EXP1 ('ΔEXP1 parasites'). One representative of n = 3 experiments is shown. (D) IFA images of condΔEXP1 trophozoites grown for 24 hours (first cycle) or 72 hours (second cycle) with and without rapalog (control) probed with α-HA to detect EXP1*-HA and α-myc for the truncated EXP1 stub in the control. Note that after excision (rapalog), the stub contains both, myc- and HA-tag, and it will be recognized by both antibodies (see panel A). Nuclei were stained with DAPI; scale bars: 5 μm. (E) Number of nuclei in DAPI-stained control and ΔEXP1 parasites (rapalog) 40 h.p.i. One representative of n = 3 independent biological replicas. (F) Long-term FC growth curve of synchronous ring control and ΔEXP1 parasites (rapalog) after depletion of EXP1 (blue arrow) at the times indicated. Blue box shows zoom of restricted to 20% parasitemia on the y-axis to show raise in the control in early time points. Orange box, PCR with primers P1 and P2 (see panel A) from gDNA of control and rapalog-treated ΔEXP1 parasites on day 9. Mean of n = 2 independent experiments. Error bars indicate SD. (G) Transmission electron microscopy images of control and ΔEXP1 parasites (rapalog) 18 to 24 h.p.i. showing hugging in ΔEXP1 [file pbio.3000473.s001.pdf]

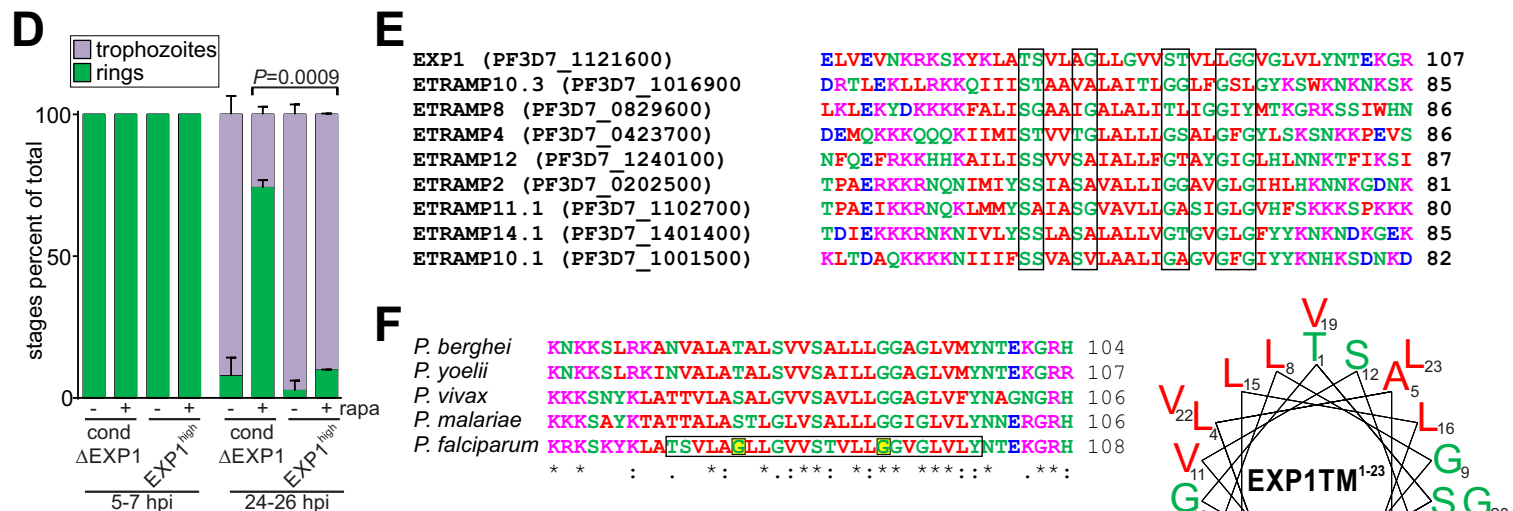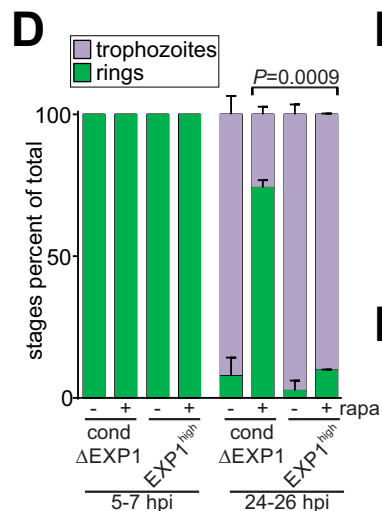

Supplement: S2 Fig — (A) Schematics of the complementation constructs expressed in condΔEXP1 parasites. Numbers refer to amino acids of the domains shown in the legend. (B) Relative activity of the complementation constructs. Except where otherwise indicated, constructs were expressed under the nmd3 (mid) promoter. Each data point (red dot) shows growth of rapalog-treated versus unexcised parasites at the end of a 5-day growth assay relative to the growth of the wt construct. Green lines indicate activity of EXP1wtnmd3 (mid) (set as 100%) and absence of activity (ΔEXP1) set as 0%; n ≥ 4 independent experiments per cell line. Error bars indicate SD. (C) Mean ± SD of relative growth versus unexcised (control) and mean of relative complementation versus EXP1wtmid (used in panel B and in the graphs in Figs 2 and 3); n numbers evident in (B). (D) Percentage of rings and trophozoites of tightly synchronous parasites of ΔEXP1 and complemented ΔEXP1 parasites at the time points indicated after invasion (after an initial cycle ± rapalog). Mean of n = 2 independent experiments. (E) Amino acid sequence of the central region (including the TM domain) of EXP1 and selected ETRAMPs from P. falciparum. Boxes show conserved G, S, and T rich regions. Hydrophobic residues, red; positively charged, pink; negatively charged, blue; polar (N, T, G, S, Q, H, and Y), green. (F) Left, alignment of the EXP1 TM region from different Plasmodium species. Asterisk, conserved and double dot, partially conserved residues; mutated G, yellow boxes; predicted TM in P. falciparum EXP1 is boxed. Right, helical wheel diagram of the PfEXP1 TM domain (numbered from 1 to 23). (PDF) [file pbio.3000473.s002.pdf]

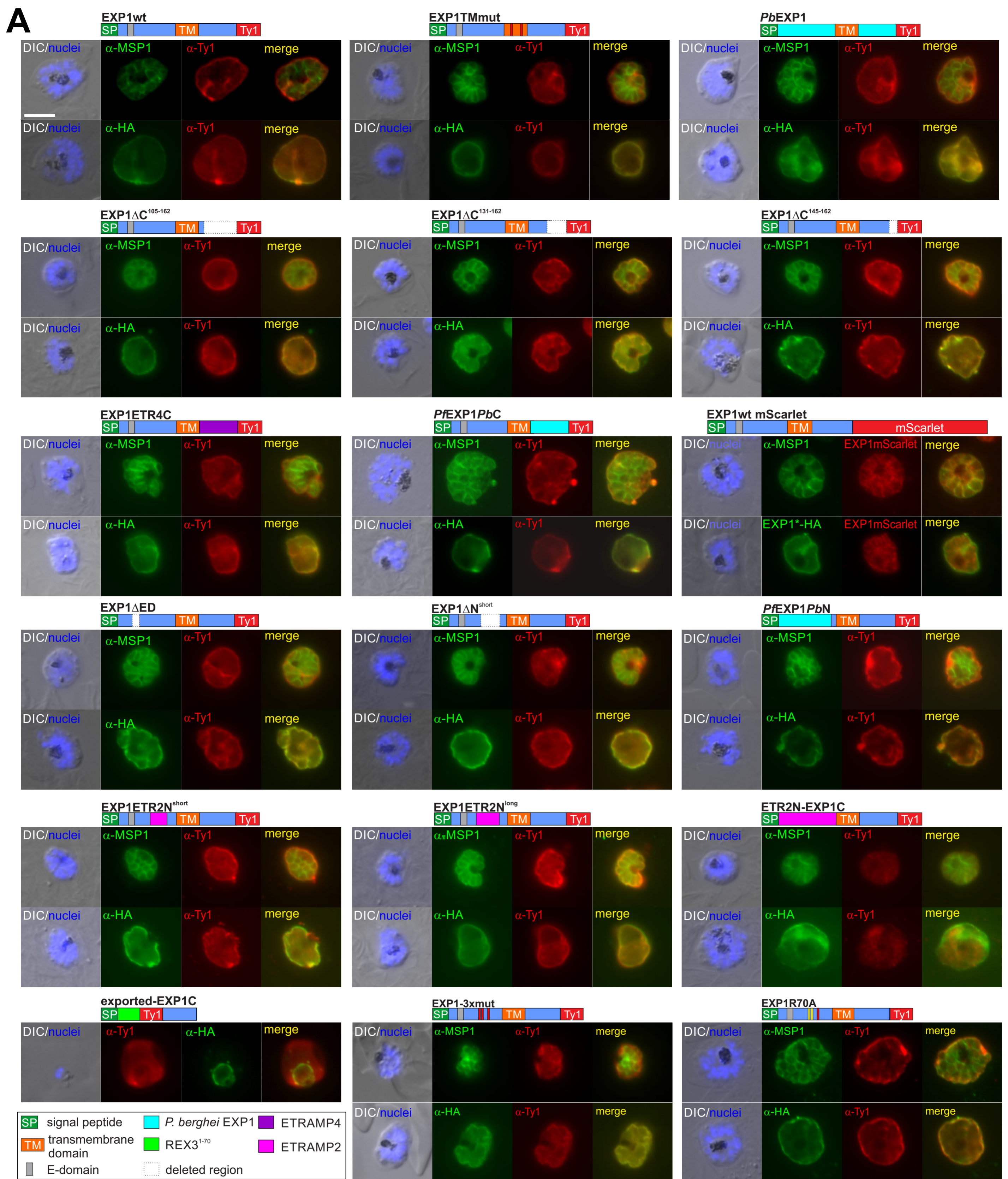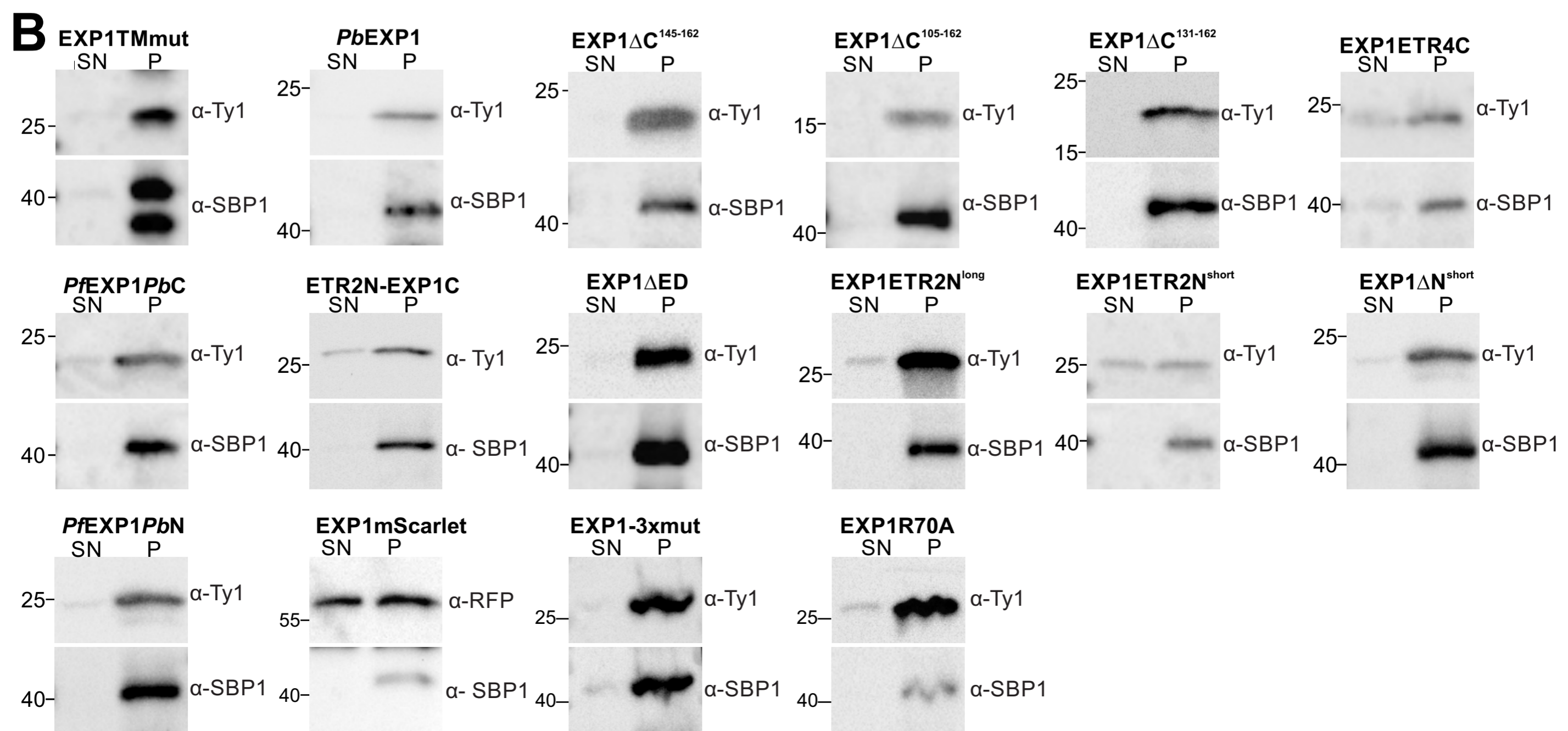

Supplement: S3 Fig — (A) IFA images of compound 2-arrested condΔEXP1 schizont stages expressing the complementation constructs indicated above each panel (α-HA detects EXP1*-HA; α-Ty1, complementing EXP1 copy; anti-RFP, EXP1mScarlet. α-MSP1, PPM). DAPI, nuclei. Scale bars: 5 μm. (B) Immunoblots of extracts of the cell lines shown in (a). Saponin was used to separate the parasite pellet (P) from the supernatant (SN) containing PV and host cell content. α-Ty1 detects the complementation constructs, anti-RFP, EXP1mScarlet and α-SBP1 was used to detect a membrane-associated control protein. DIC, differential interference contrast. (PDF) [file pbio.3000473.s003.pdf]

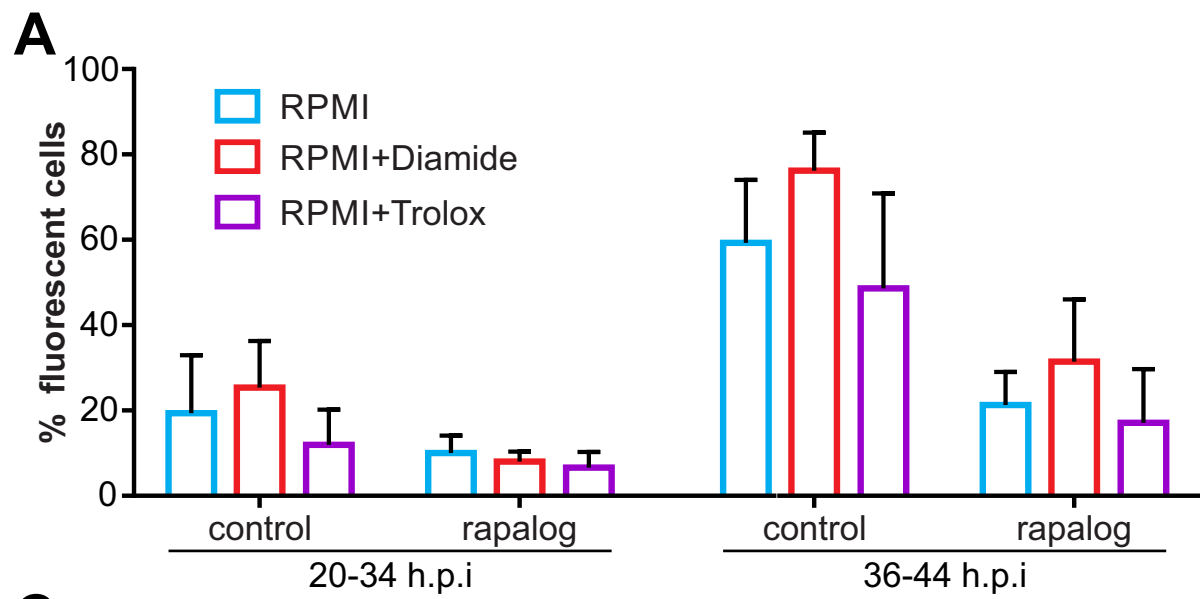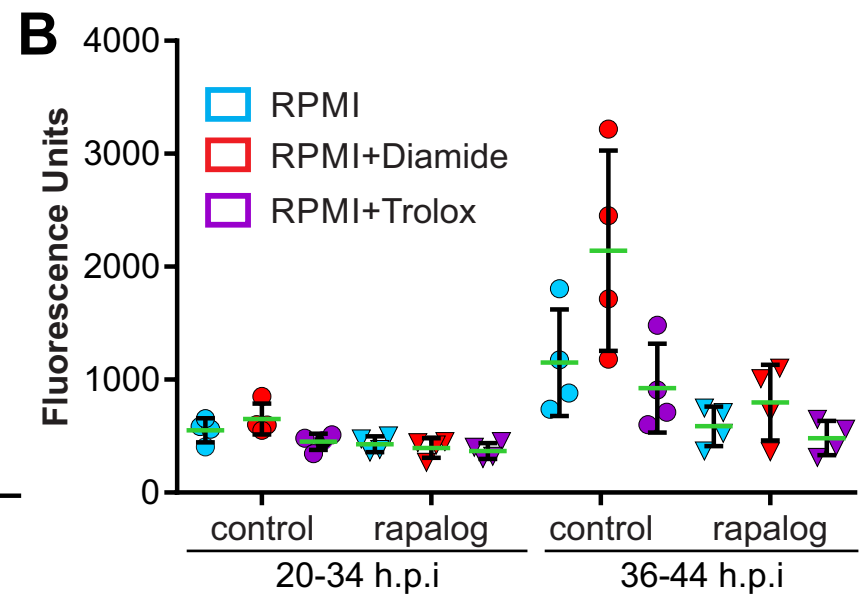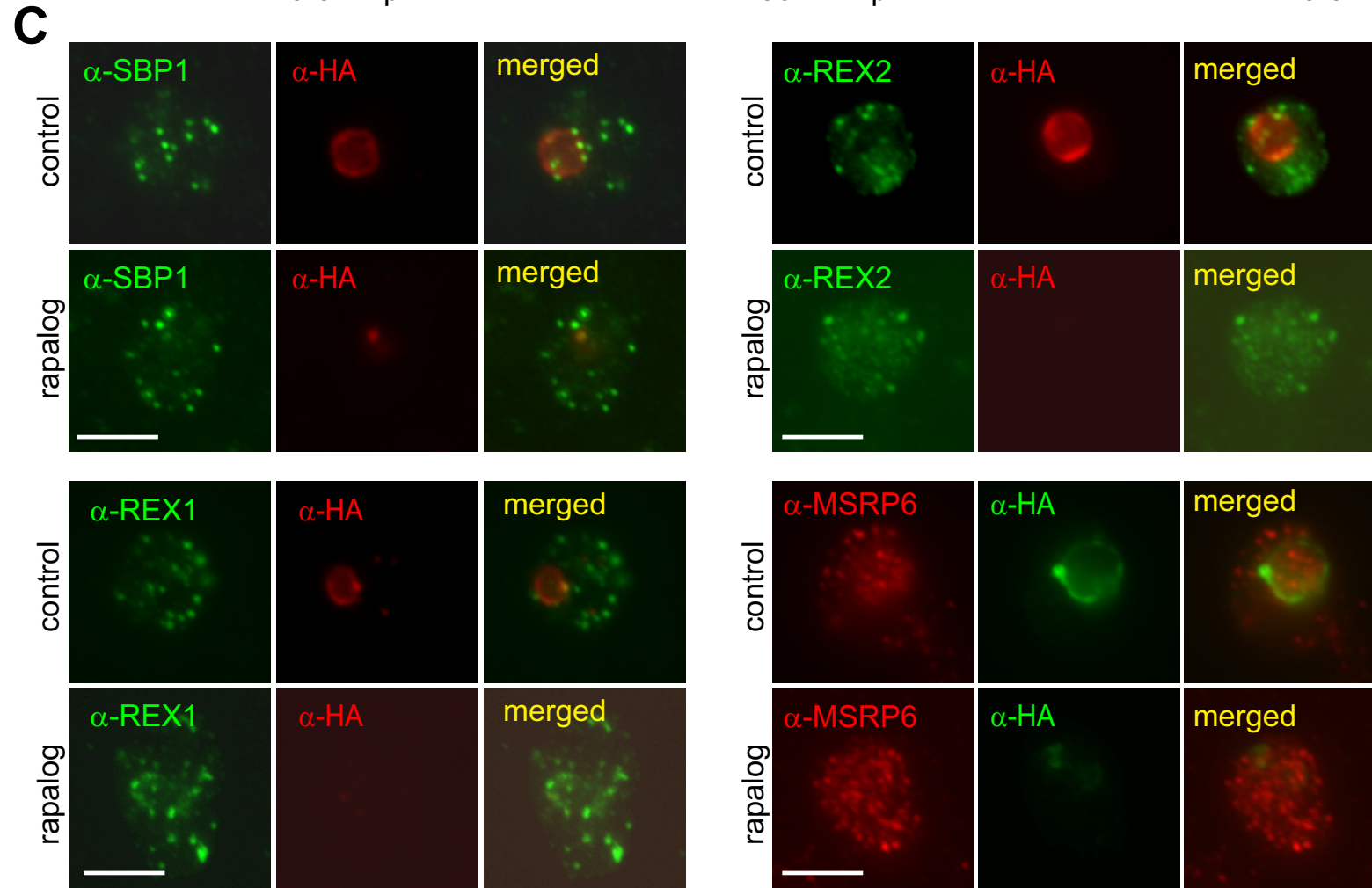

Supplement: S4 Fig — (A) FC analysis of matching 5-ALA-treated control and ΔEXP1 parasites (rapalog) after incubation with CM-H2DCFDA in RPMI alone or supplemented with diamide or Trolox at the time points indicated. The percentage of cells with oxidative stress corresponds to the number of CM-H2DCFDA positive cells of the total number of 5-ALA-positive cells. Error bars, SD. n = 4 independent biological replicas. (B) Fluorescence of control and ΔEXP1 parasites analyzed in (a). Green line, mean; error bars, SD. n = 4 independent biological replicas. (C) IFA images of control and ΔEXP1 parasites (rapalog) probed with α-HA (EXP1*-HA), α-SBP1, α-REX1, α-REX2, and α-MSRP6. Size bars, 5 μm. h.p.i., hours post invasion. (PDF) [file pbio.3000473.s004.pdf]

**A****cond $\Delta$ EXP1 + EXP2-GFP (live)**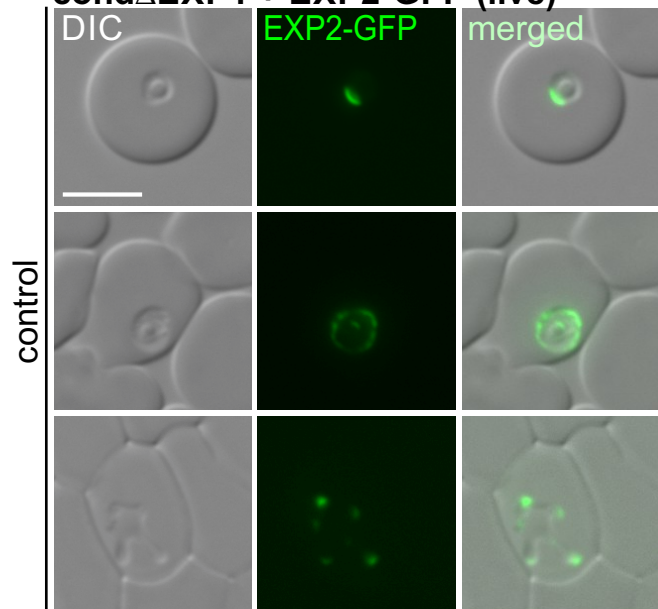

rapalog

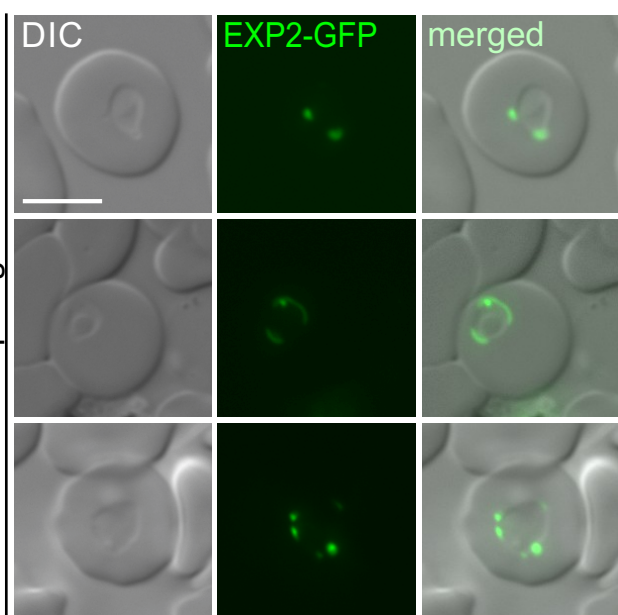**B****cond $\Delta$ EXP1 (IFA)**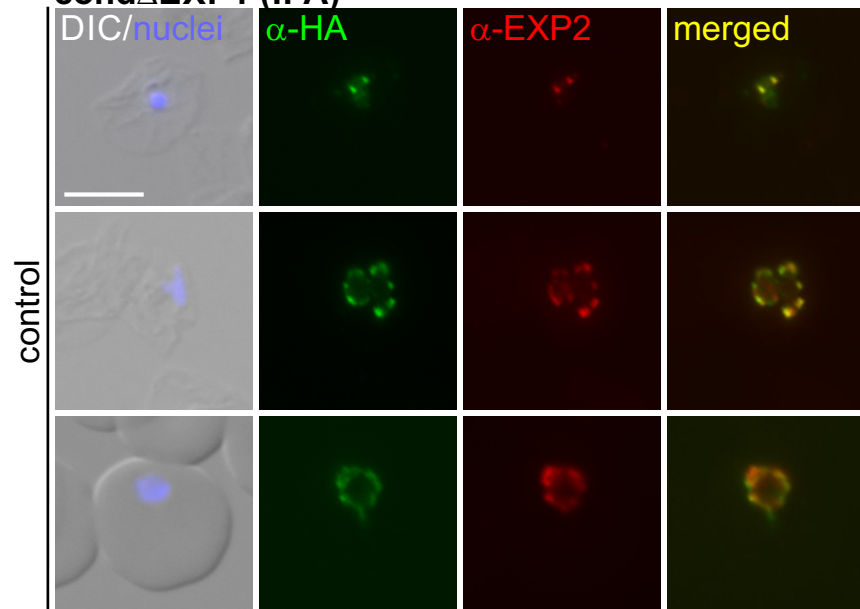

rapalog

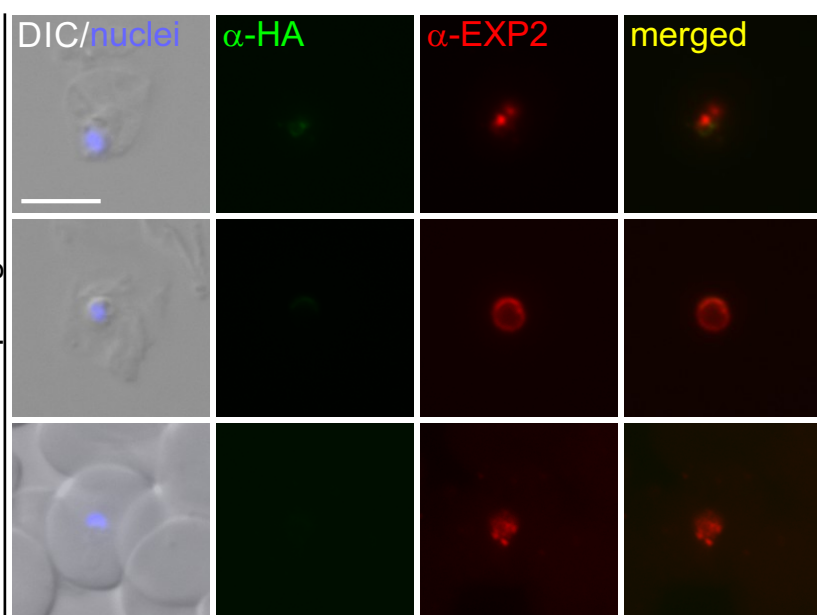

Supplement: S5 Fig — (A) Live cell images of control and ΔEXP1 (rapalog) ring stages episomally expressing EXP2-GFPnmd3. (B) IFA images of control and ΔEXP1 ring stages (rapalog); α-HA detects EXP1*-HA, α-EXP2 detects endogenous EXP2. Nuclei were stained with DAPI. Scale bars: 5 μm. DIC, differential interference contrast. (PDF) [file pbio.3000473.s005.pdf]

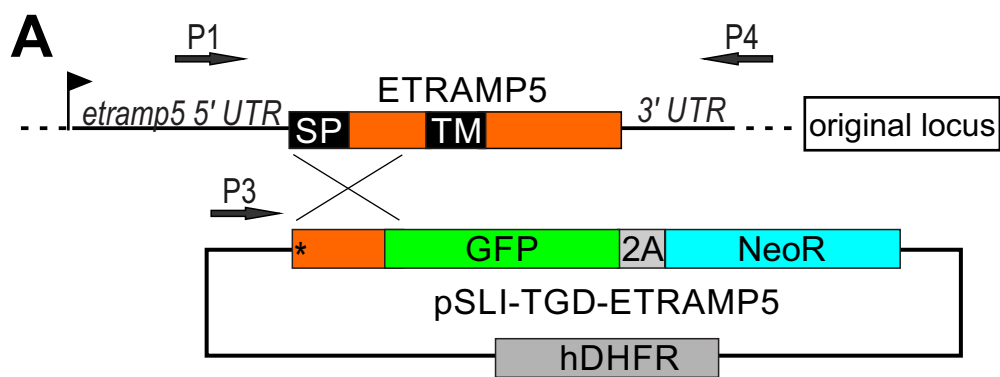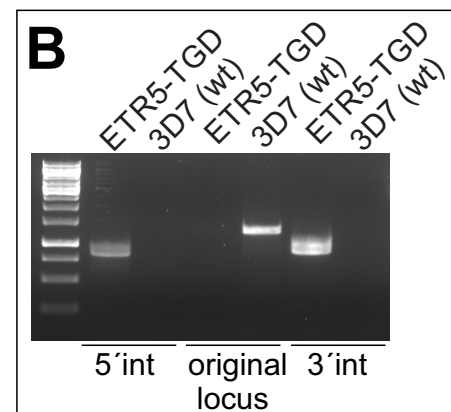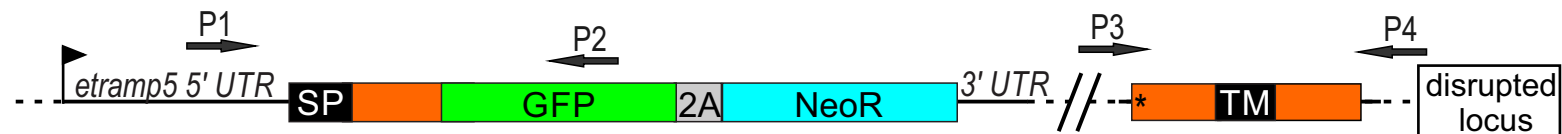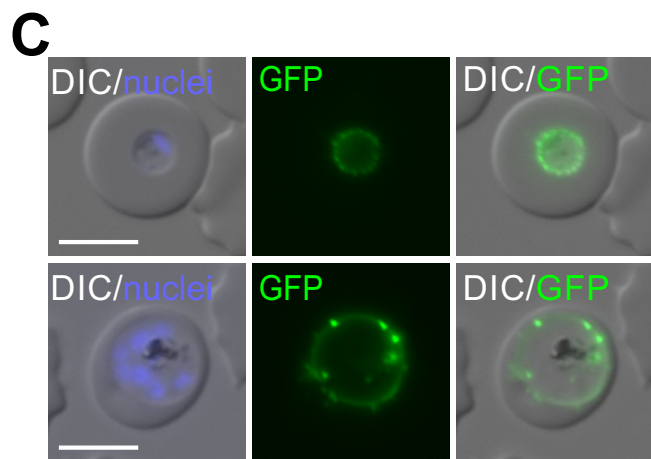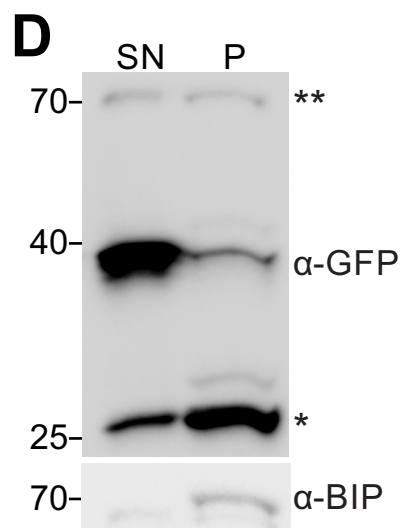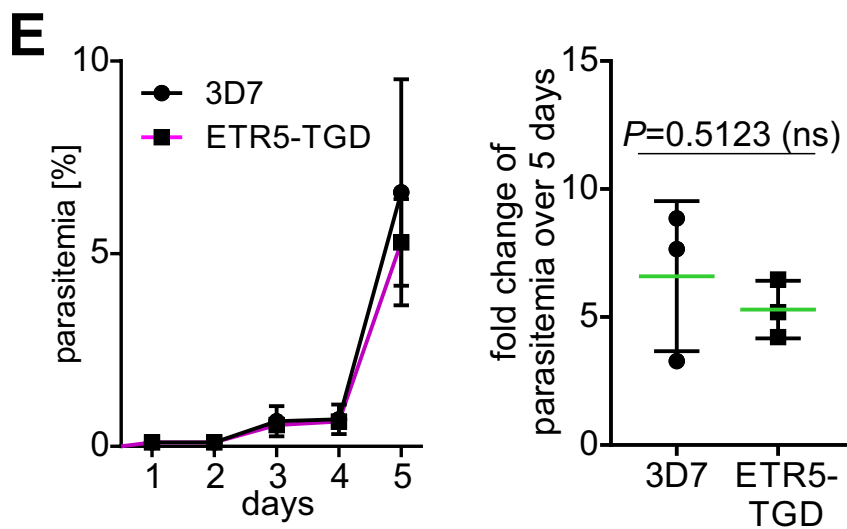

Supplement: S6 Fig — (A) Schematic representation of SLI-TGD to disrupt etramp5. Features as in S1A Fig. (B) PCR on gDNA of ETR5-TGD and wild-type 3D7 parasites confirming: 5'Int, 5'integration; absence of 'original locus'; 3'Int, 3' integration. (C) Live cell images of young trophozoite and schizont stages of ETR5-TGD parasites (fluorescence shows the truncated GFP-tagged protein). DAPI, nuclei; scale bars: 5 μm. (D) Immunoblot of extracts of ETR5-TGD parasites separated into saponin supernatant (SN, containing PV and host cell content) and parasite pellet (P). α-GFP, detects truncated ETR5; α-BIP: control for the parasite pellet. Asterisk, protein degraded down to GFP; double asterisk, unskipped protein (first T2A, no unskipped product detected at the GFP-Neomycin junction). The truncated protein has no TM and is therefore found in the SN. (E) Left: FC 5-day growth curves of synchronous 3D7 and ETR5-TGD parasites. Mean of n = 3 independent biological replicas. Right: fold increase in parasitemia over 5 days for 3D7 and ETR5-TGD parasites measured by FC. Green line indicates mean and error bars SD, two-tailed unpaired t test; P value indicated. DIC, differential interference contrast; ns, not significant. (PDF) [file pbio.3000473.s006.pdf]

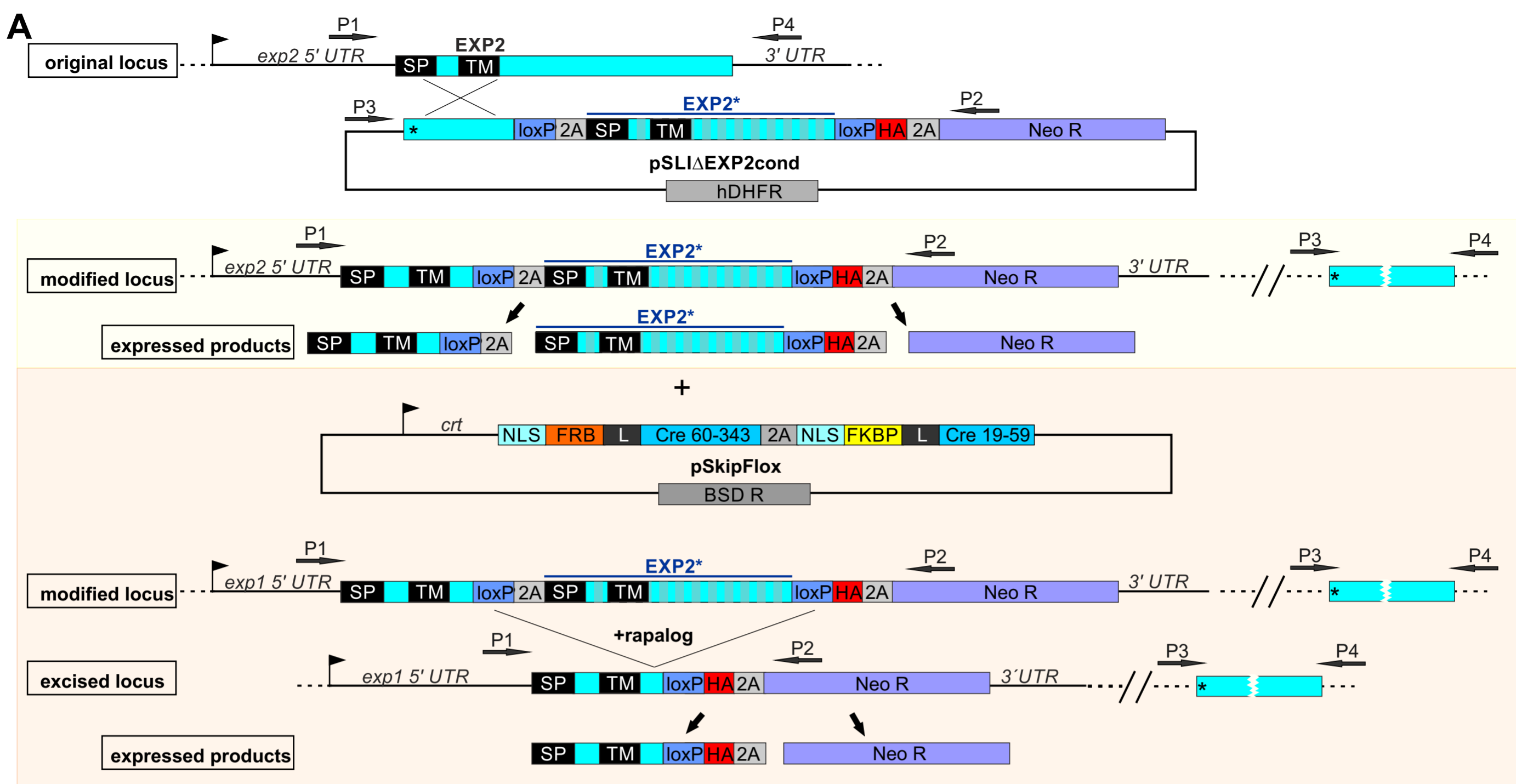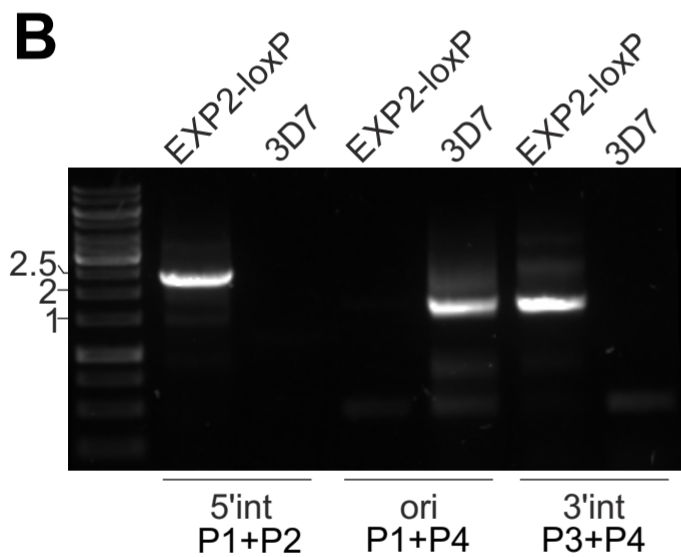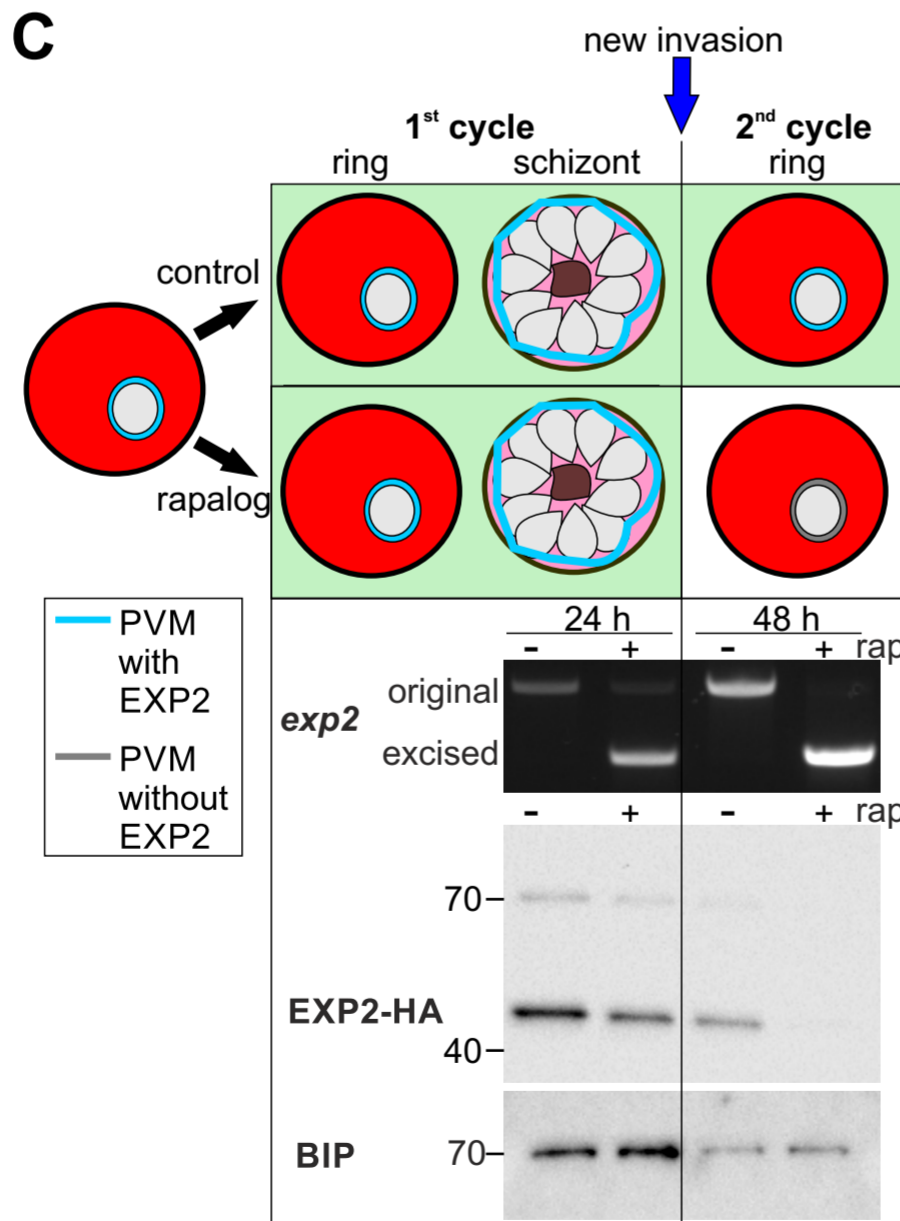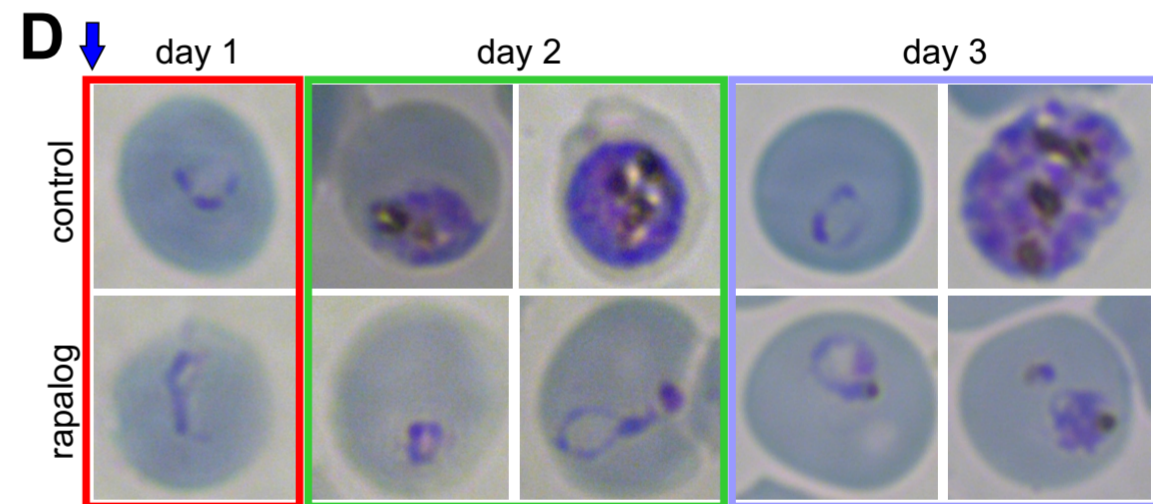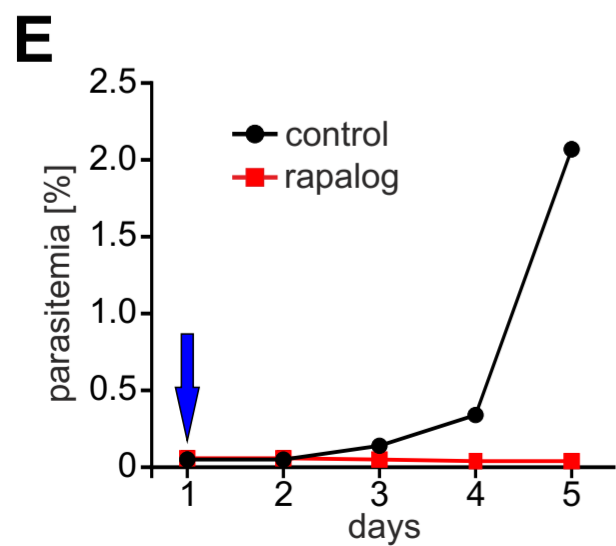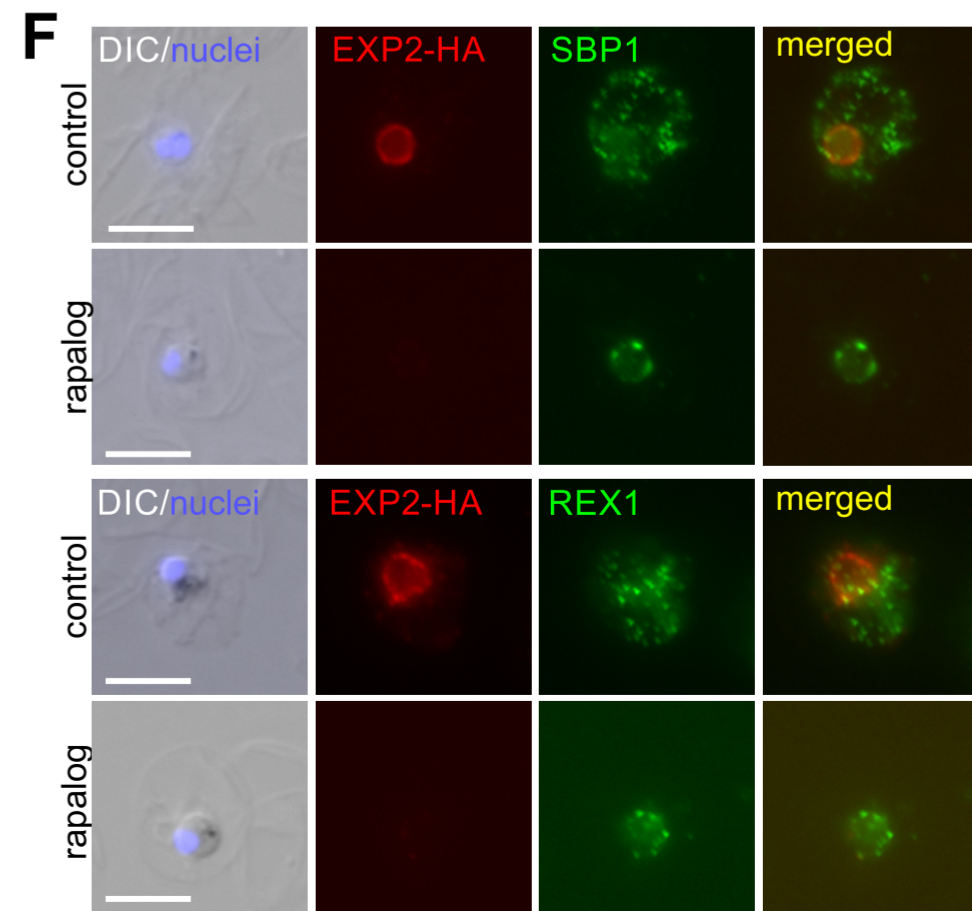

Supplement: S7 Fig — (A) Schematic representation of the SLI strategy to obtain a cell line for DiCre-based conditional KO of exp2. Features as in S1A Fig. (B) PCR products from gDNA of condΔEXP2 and wild-type 3D7 parasites confirming: 5'Int, 5'integration; absence of 'original locus'; 3'Int, 3' integration. (C) Strategy to deplete EXP2 from the PVM using synchronized condΔEXP2 ring stages divided into a culture with and one without rapalog. Top: schematic: green boxes and blue line around the parasite signify PVM with EXP2. Mid: PCR with primers P1 and P2 from gDNA 24 hours and 48 hours after addition of rapalog. Original: PCR product for locus with intact exp2; excised: PCR product after excision of exp2. Bottom: western blot using α-HA to detect EXP2*-HA and α-BIP as loading control. Note that the small truncated fragment after excision of the functional copy becomes HA-tagged (see panel A) but is not detected (likely due to its small size and its instability leading to low abundance, see panel F). (D) Giemsa smears of synchronous ΔEXP2 parasites (rapalog) compared to the controls. Blue arrow indicates start of a new cycle without EXP2. (E) FC growth curves of synchronous ring stage condΔEXP2 parasites grown ± rapalog over 5 days. Blue arrow indicates start of cycle without EXP2. One representative of n = 3 experiments. (H) IFA images of control and ΔEXP2 parasites (rapalog) probed with α-HA, which detects full functional (control) or truncated inactivated (rapalog) EXP2-HA and SBP1 (α-SBP1) or REX1 (α-REX1). DAPI, nuclei. Scale bars: 5 μm. Note that the truncated inactive version of EXP2 is not well detected, likely because it is degraded. DIC, differential interference contrast. (PDF) [file pbio.3000473.s007.pdf]

**A**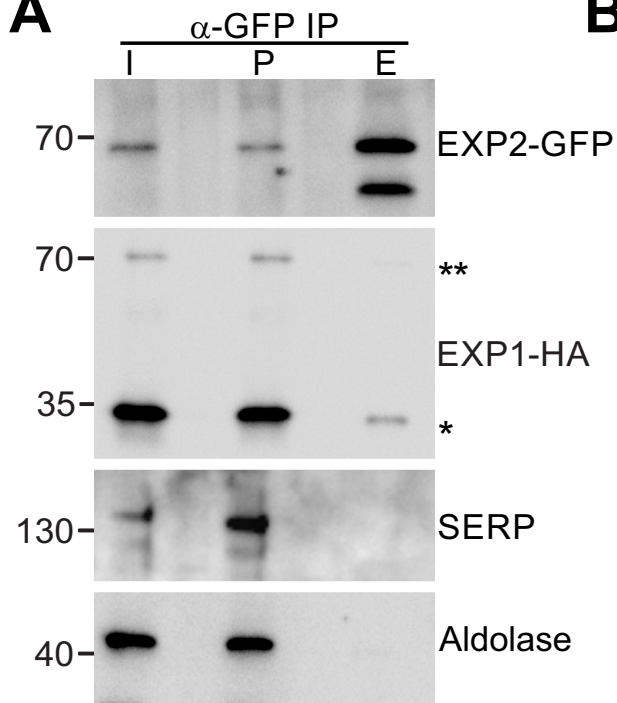**B**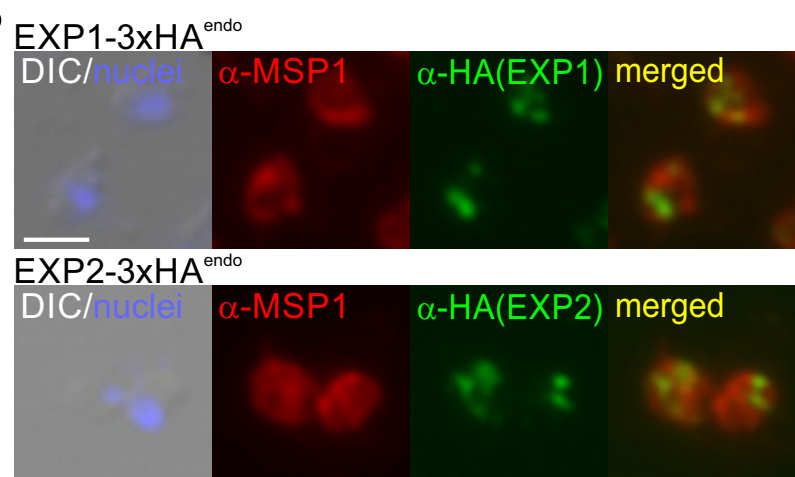**C**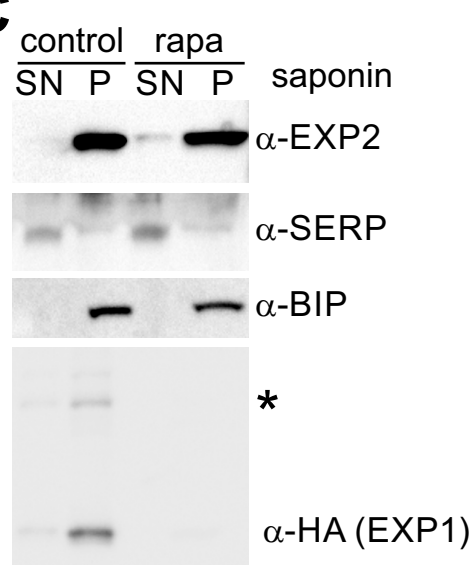**D**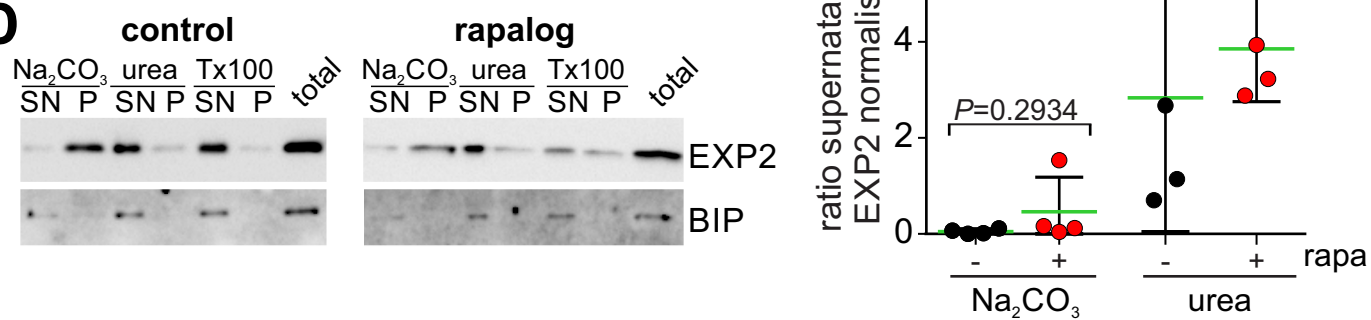**E**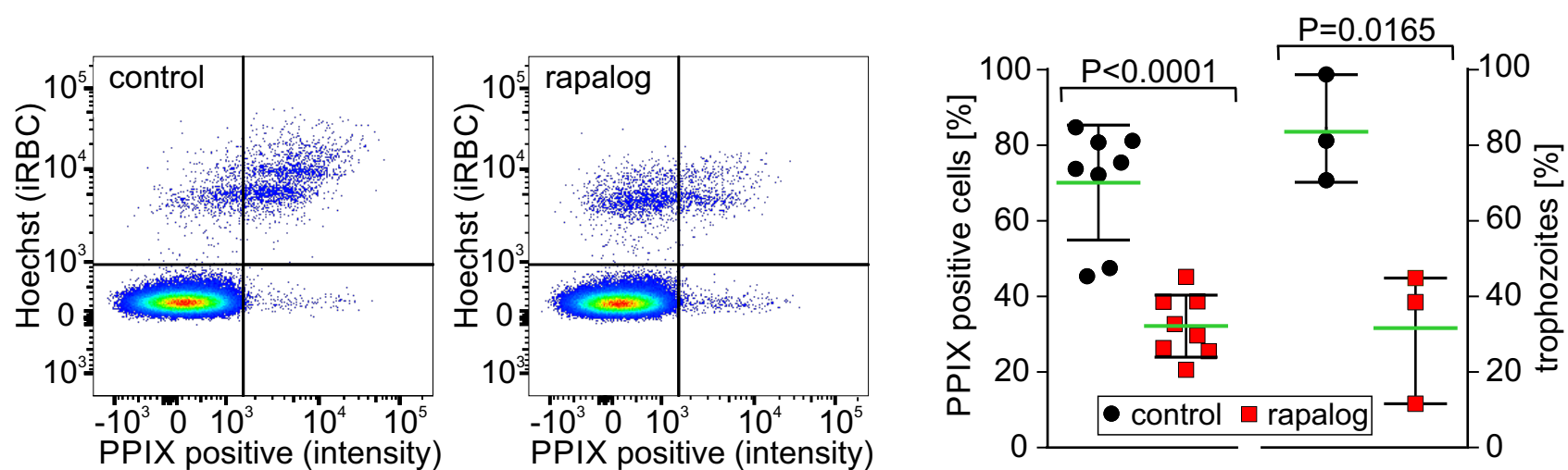**F**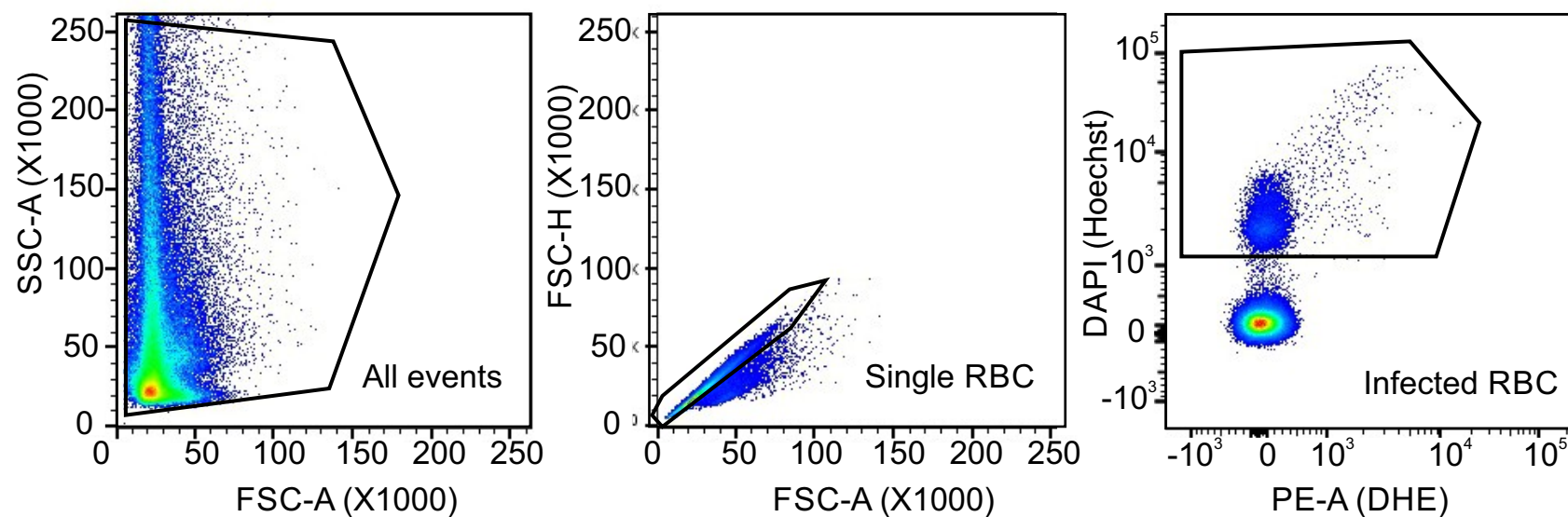

Supplement: S8 Fig — (A) Western blot of reciprocal co-IP experiment using α-GFP with extracts of the cell line condΔEXP1+EXP2-GFPnmd3 to pull down EXP2-GFP. α-HA detects EXP1*-HA (monomer: asterisk, dimer: double asterisk); α-SERP, soluble PV protein; α-aldolase, cytosolic parasite protein. Input (I): total lysate before IP; post IP (P): lysate after IP; Eluate (E). One representative of n = 3 independent experiments. (B) IFA images of EXP1-3xHAendo and EXP2-3xHAendo merozoites probed with α-HA and α-MSP1 (plasma membrane marker). Nuclei were stained with DAPI; scale bar 2 μm. (C) Immunoblot of protein extracts derived from ΔEXP1 (rapa) and control trophozoites. Saponin was used to separate parasite pellet (P) from the supernatant (SN) containing PV and host cell soluble proteins. α-EXP2 detects endogenous EXP2; α-SERP, a soluble PV soluble to control for proper PVM permeabilization; α-BIP, as loading control and α-HA (detecting EXP1*-HA) to show loss of EXP1. One representative of n = 2 experiments. (D) Left: immunoblot of protein extracts from ΔEXP1 (rapa) and control trophozoites fractionated in SN and P after hypotonic lysis and extraction with Na2CO3, urea (peripheral membrane proteins) and Triton x-100 (TX-100, integral membrane proteins). α-EXP2 detects endogenous EXP2 and α-BIP, a parasite-internal peripheral membrane protein. Right: densitometric analysis of EXP2 intensity in SN and P. The ratio SN/ P of the EXP2 signal was calculated for Na2CO3 and urea and normalized to the ratio of BIP. Green line: mean of n = 4 independent experiments; error bars, SD. P values were calculated with a two-tailed unpaired t test. (E) FC analysis of 5-ALA–treated control and ΔEXP1 parasites (rapalog) shows that the number of PSAC-positive cells correlates with the number of cells that reached the trophozoite stage, irrespective of whether parasites contained EXP1 or not. Left, gating for Hoechst/PPIX-positive cells (upper right quadrant). Right: quantification of PPIX-positive cells and percen [file pbio.3000473.s008.pdf]
