# Supplementary material for: EXP1 is critical for nutrient uptake across the parasitophorous vacuole membrane of malaria parasites
Source: PLoS Biol. 2019 Sep 30;17(9):e3000473. doi: 10.1371/journal.pbio.3000473 (PMC6786648; doi:10.1371/journal.pbio.3000473)
Supplement: S1 Table — (PDF) [file pbio.3000473.s009.pdf]

Table S1. Oligonucleotides used in this study for cloning and confirming correct integration of plasmid constructs into the genome (restriction sites are indicated in lower case and overlapping regions in bold).

Oligonucleotide	Sequence (5`-3`)	Construct
EXP1 (N) NotI fw	**GGTGACACTATAGAATACTC**gcggccgcCCGGTAAAAAATCTTATCAGTATTTTTTCTTGCTC	pSLI-*exp1-loxP*
EXP1(N) rv SpeI	**TAAATCTTCTTCGCTTATGAG**TTTTTGTTCACCCAAATCCTCTTCTGATATTAACTTCTGCTCactagtATCAGATATTAAATCGTGTACATC	
myc-loxP-T2A rv2	**TTCAACATCACCACAAGTCAAC**AAAGAACCTCTACCTTCACCGGATAACTTCGTATAATGTATGCTATACGAAGTTAT**TAAATCTTCTTCGCTTATGAG**	
EXP1 recod T2A fw1 AvrII	**GTTGACTTGTGGTGATGTTGAA**GAAAATCCAGGTCCAcctaggATGAAGATACTTAGTGTTTTCTTC	
EXP1 recod loxP-3XHA rv XmaI Gibson	CTGGAACATCGTATGGGTACATGGTggtaccGGATAACTTCGTATAATGTATGCTATACGAAGTTATcccgggATGCTCTGTTCCTGAAACTAAATTATTATC	
EXP2 NotI fw Gibson	**CTATTTAGGTGACACTATAGAATACTC**gcggccgcCCGGTAAAAAGTCAGTTATATATTTTCCTTTTTTTT	pSLI-*exp2-loxP*
EXP2 rv SpeI	ACTAGTTTGTCTTAATTTTGTGGCAAAGTTGTTTCTG	
2A- EXP2 recod fw 1	**TACGAAGTTATCCGGTGAAGGTAGAGGTTCTTTG**TTGACTTGTGGTGATGTTGAAGAAAATCCAGGTCCAATGAAGGTGTCCTACATTTTCTCTTTC	
loxP-2A EXP1 fw2	CAGAAACAACTTTGCCACAAAATTAAGACAAactagtATAACTTCGTATAGCATACATTA**TACGAAGTTATCCGGTGAAGGTAGAGGTTCTTTG**	
EXP2 recod rv –loxP XmaI Gibson	**CGTATAATGTATGCTATACGAAGTTAT**cccgggCTCCTTGTTCTCGTCCTTCTTCTCG	
EXP1 rec Xho fw	CTCGctcgagATGAAGATACTTAGTGTTTTCTTCTTAGC	pEXP1comp ^{nmd3,hsp86,sfa32} EXP1wt-Ty1 EXP1$^{1\text{-}162}$ For all EXP1 complementation constructs
EXP1 full length TY1 rv1	**CCTGATTTGTATGGACTTCGGCGTCC**AGGGGGTCCTGGTTGGTGTGCACCTCcctaggATGCTCTGTTCCTGAAACTAAATTATTATC	
Ty1 rv2	TCCTcccgggTTAAGCGTCCAGGGGATCCTGATTGGTGTGCACTTCGGCATCCAGAGGAT**CCTGATTTGTATGGACTTCGGCGTCC**	
EXP1 rec rv AvRI	TCCTcctaggATGCTCTGTTCCTGAAACTAAATTATTATC	
EXP1ΔC (105-162) rv AvrII	TCCTcctaggCTTCTCTGTGTTATAAAGAAC	pEXP1comp ^{nmd3} EXP1ΔC$^{105\text{-}162}$ Ty1
EXP1ΔC (131-162) rv AvrII	TCCTcctaggACCGTTACTCTCGCTGTCTGCGTCAGG	pEXP1comp^{nmd3} EXP1ΔC$^{131\text{-}162}$Ty1
EXP1ΔC (145-162) rv AvRII	TCCTcctaggAGTTACGTCCTGTGCAGTTACC	pEXP1comp ^{nmd3} EXP1ΔC$^{145\text{-}162}$Ty1
EXP1 mut TM rv1	**GAACAAGTCCTACTCCTAAAAG**AAGAACTGTACTAACAACTCCAAGAAGTAAAGCTAAAACACTTGTTGCTAACTTGTACTTACTCTTACG	pEXP1comp^{nmd3} EXP1TMmutTy1

Oligonucleotide	Sequence (5`-3`)	Construct
EXP1 mut TM fw2	**CTTTTAGGAGTAGGACTTGTTC**TTTATAACACAGAGAAGGGTCGTCATCC	G83L G94L
EXP1 rv1 3x mut	**CAGCTAAAACACTTGTT**GCTAACTTGTACTTACTCTTGGCCTTATTTACTGCTGCTAACTCCTCCTCCTTCTTTATC	pEXP1comp ^{nmd3} EXP1-3xmutTy1 V65A E66A R70A
EXP1 fw2	**CAAGTTAGCAACAAGTGTTTTAGCTG**GACTTCTTGGAGTTGTTAGTACAGTTC	
EXP1 rv1 R70A	**CAGCTAAAACACTTGTTG**CTAACTTGTACTTACTCTTGGCCTTATTTACCTCTACTAACTCCTCCTCCTTCTTTATC	pEXP1comp^{nmd3,hsp86} EXP1R70ATy1
EXP1 fw2	**CAAGTTAGCAACAAGTGTTTTAGCTG**GACTTCTTGGAGTTGTTAGTACAGTTC	
EXP1+ETRAMP4(C) rv1	**GTTTTTATTAGATTTACTAAG**CTTCTCTGTGTTATAAAGAACAAGTCCTACTC	pEXP1comp^{nmd3} EXP1ETR4(C)Ty1 EXP1$^{1-105}$ ETRAMP4$^{76-136}$
ETRAMP4 (C) fw2	**CTTAGTAAATCTAATAAAAAAC**CAGAAGTATCAGGTGACGAAAAAG	
ETRAMP4 (C) rv2 AvRII	TCCTcctaggAACAGTAGTGGGTACTGCTGTAG	
P. berghei EXP1 fw Xho I	CTCGctcgagATGAAAATCAATATAGCTTCAATTC	pEXP1comp^{nmd3} *Pb*EXP1Ty1 *Pb*EXP1$^{1-166}$
P. berghei EXP1 AvRII rv	TCCTcctaggTTGTTGAAGATTTGGCATGTTAAGTGG	
EXP1 P. f + P.b EXP1 C rv1	**CCAATTTGGAATGGATGTCTTCC**CTTCTCTGTGTTATAAAGAACAAGTC	pEXP1comp^{nmd3} *Pf* EXP1 *Pb* (C) Ty1 *Pf* EXP1 $^{1-105}$ *Pb*EXP1 $^{98-166}$
P.b EXP1 (C) fw2	**GGAAGACATCCATTCCAAATTGG**CAAATCAGAAAGGGGAACATCTG	
P. b EXP1 (N)+ EXP1 P.f TM rv1	**GAAGTCCAGCTAAAACACT**TGTTGCTAACTTGTTTGCTTTTCTTAAAGATTTTTTATTTTTTG	pEXP1comp ^{nmd3} EXP1*Pb*(N)Ty1 *Pb*EXP1$^{1-74}$ *Pf*EXP1$^{88-162}$
EXP1 P. f TM fw2	**AGTGTTTTAGCTGGACTTCTTGG**	
REX3 fw XhoI	CTCGctcgagATGCAAACCCGTAAATATAATAAGATG	pEXP1comp^{nmd3} Exported EXP1CTy1 REX3$^{1-70}$-Ty1-EXP1$^{102-162}$
REX3 1-70 + Ty1 rv 1	**CCTGATTTGTATGGACTTCGGCGT**CCAGGGGGTCCTGGTTGGTGTGCACCTCCCTAGGTGCTTCTATATGTGATGACTCTGC	
Ty1 + EXP1 (C) fw2	**ACGCCGAAGTCCATACAAATCAGG**ATCCTCTGGATGCCGAAGTGCACACCAATCAGGATCCCCTGGACGCTggtaccAACACAGAGAAGGGTCGTCATC	
EXP1 (C) rv XmaI	TCCTcccgggTTAATGCTCTGTTCCTGAAACTAAATTATTATC	
EXP1 + ETRAMP2 (N) long rv1	**GGTGTTAATTTTTTGGCTGGCTTTGC**TTCTACCATGCTATTGTAATCTCTTGGACTACCCTTCTTGTTCTTCTTCTTTG	pEXP1comp^{nmd3} EXP1ETR2(N)longTy1 EXP1$^{1-43}$ ETRAMP2$^{21-50}$ EXP1$^{74-162}$
ETRAMP2 (N) long + EXP1 fw2	**GCAAAGCCAGCCAAAAAATTAACACC**AGCAGAAAGGAAAAAGAGAAATCAAAATATAATGATATACAAGTTAGCAACAAGTGTTTTAG	
EXP1 + ETRAMP2 N short rv1	**GATTTCTCTTTTTCCTTTCTG**CTGGTGTTAATATCATGTCGCTAATAAGGTCATG	pEXP1comp^{nmd3} EXP1ETR2(N)shortTy1 EXP1$^{1-58}$ ETRAMP2$^{36-50}$ EXP1$^{74-162}$
ETRAMP2 N short + EXP1 fw2	**CAGAAAGGAAAAAGAGAAATC**AAAATATAATGATATACAAGTTAGCAACAAGTGTTTTAGC	
EXP1 ΔN rv	**GCTAACTTGTACTTACTCTT**CTTCTTTATCATGTCGCTAATAAGG	pEXP1comp^{nmd3} EXP1ΔN-Ty1

Oligonucleotide	Sequence (5`-3`)	Construct
EXP1 fw ΔN	**AAGAGTAAGTACAAGTTAGC**	Δ61-70
EXP1 fw1 (w/o E domain)	**CATAATATTTAACAAGGAGA**GTCTTGCAGAGAAGACTAATAAGGGTACAGGTGGTAGTGGAGAGCCTCTTATTGACG	pEXP1comp^{nmd3} EXP1ΔEDTy1 ΔEDEXP1$^{31-41}$
EXP1 fw2 (w/o E domain)	CTCGctcgagATGAAGATACTTAGTGTTTTCTTCTTAGCATTATTTTT**CATAATATTTAACAAGGAGA**	
ETRAMP2 XhoI fw	CTCGctcgagATGAAACTCTCCAAAATCTTATATTTCTTCG	pEXP1comp^{nmd3} ETR2N-EXP1(C)Ty1 ETRAMP2$^{1-77}$ EXP1$^{102-162}$
ETRAMP2 rv1 EXP1 C	**GGATGACGACCCTTCTCTGTGTT**CTTGTTATTTTTATGTAAATGAATAC	
EXP1 C-terminus fw 2	**AACACAGAGAAGGGTCGTCATCC**TTTTAAGATTGGTAGTTCAGATCC	
SBP1 XhoI fw Gibson	ATAACGTATATCATTTTAAAGATAActcgagATGTGTAGCGCAGCACGAGCATTTG	pmScarlet^{nmd3} SBP1mScarlet
mScarlet fw AvrII	cctaggATGGTGAGTAAGGGTGAGGCAGTG	
SBP1-mScarlet rv AvrII	CACTGCCTCACCCTTACTCACCATcctaggGGTTTCTCTAGCAACTGTTTTTGTTGTGG	
mScarlet rv XmaI Gibson	CATTAAGCTGCCATATCCCTCGAcccgggTTACTTGTAAAGCTCATCCATACCACC	
EXP1 rec Xho fw	CTCGctcgagATGAAGATACTTAGTGTTTTCTTCTTAGC	pmScarlet^{nmd3} EXP1mScarlet
EXP1 rec rv AvRI	TCCTcctaggATGCTCTGTTCCTGAAACTAAATTATTATC	
PF13_0191 fw XhoI	CTCGctcgagATGCAAAGTGAATTCTTCATTTG	pmScarlet^{nmd3} SPmScarlet PF13_0191 $^{1-73}$
PF13_0191 rv AvrII	TCCTcctaggGTCTACCTTTTTTTTTAATAATTC	
EXP2-XhoI fw Gibson recodonised	**CGTATATCATTTTAAAGATAA**ctcgagATGAAGGTGTCCTACATTTTCTCTTTC	pGFP^{nmd3} EXP2-GFP
EXP2-KpnI rv Gibson)	ggtaccCTCCTTGTTCTCGTCCTTCTTCTCG	
EXP2-GFP fw Gibson	**CGAGAAGAAGGACGAGAACAAGGAGggtacc**ATGAGTAAAGGAGAAGAACTTTTCAC	
GFP rv XmaI Gibson	**CATTAAGCTGCCATATCCCTCGA**cccgggTTATTTGTATAGTTCATCCATGCCATG	
ETRAMP5 NotI fw TGD	CTCGgcggccgcTAAAGATTCTCCAAAGTATTTTCTTTTTTCG	pSLI-ETRAMP5TGD
ETRAMP5 MluI rv TGD	TCCTacgcgtTGAAGATGAGGAAGCTGGGGATGATG	
EXP1 NotI fw TGD	CTCGgcggccgcTAAAAAATCTTATCAGTATTTTTTCTTGCTC	pSLI-EXP1TGD
EXP1 MluI rv TGD	TCCTacgcgtTACTACACCTAATAAACCTGCAAGTACTG	
