# Supplementary material for: EXP1 is critical for nutrient uptake across the parasitophorous vacuole membrane of malaria parasites
Source: PLoS Biol. 2019 Sep 30;17(9):e3000473. doi: 10.1371/journal.pbio.3000473 (PMC6786648; doi:10.1371/journal.pbio.3000473)
Supplement: S1 Raw images — (PDF) [file pbio.3000473.s012.pdf]

Figure 1C upper panel

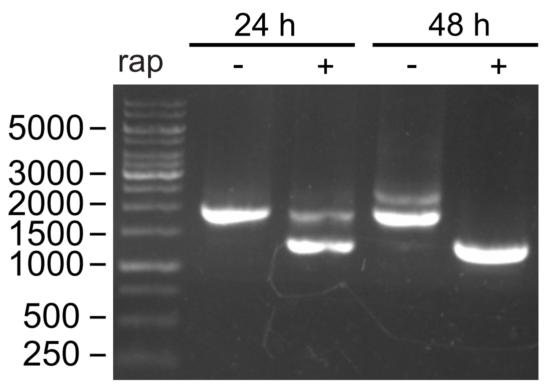

Figure 1C lower panel

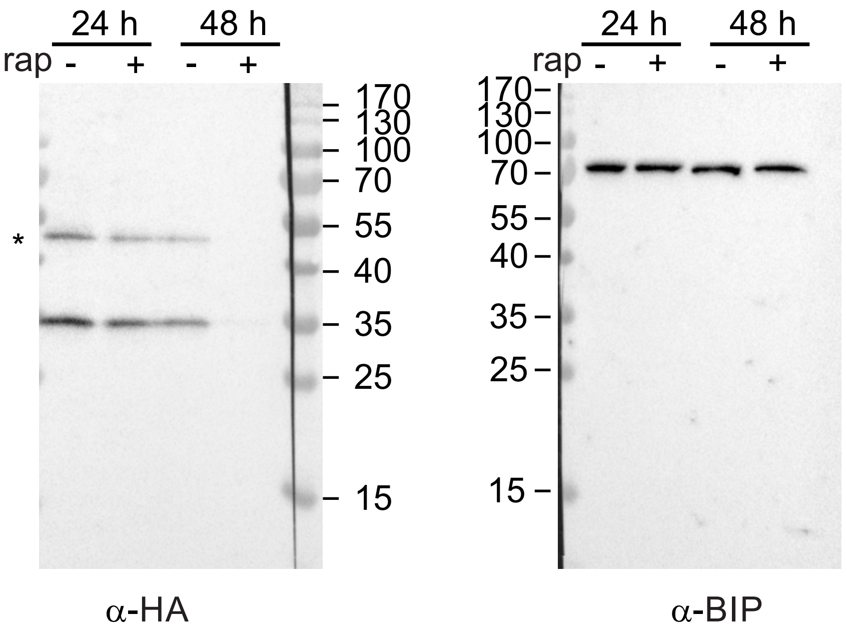

Figure 2F right panel

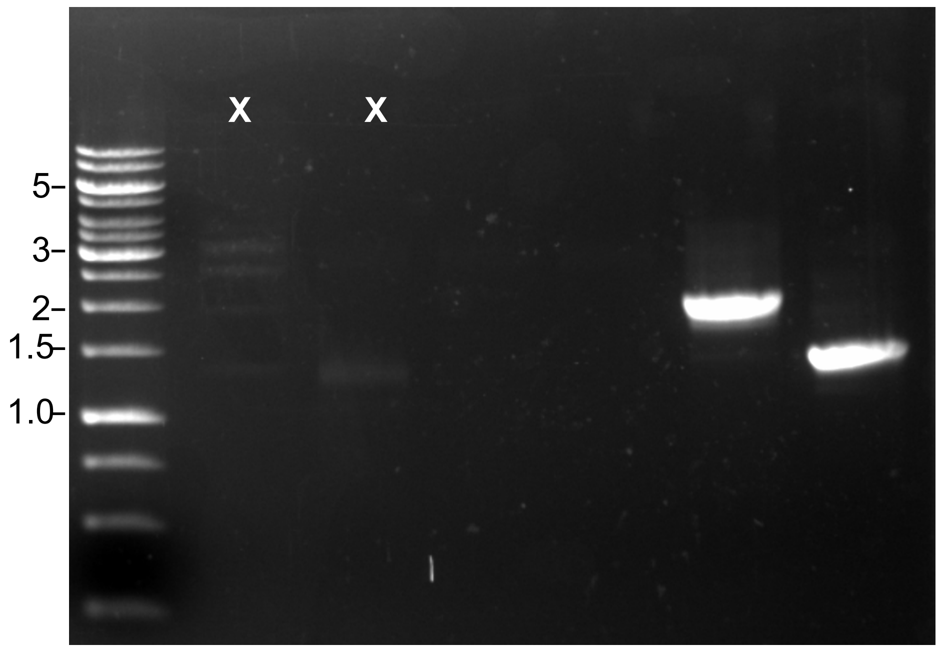

X: Samples not corresponding to figure

Figure 3A

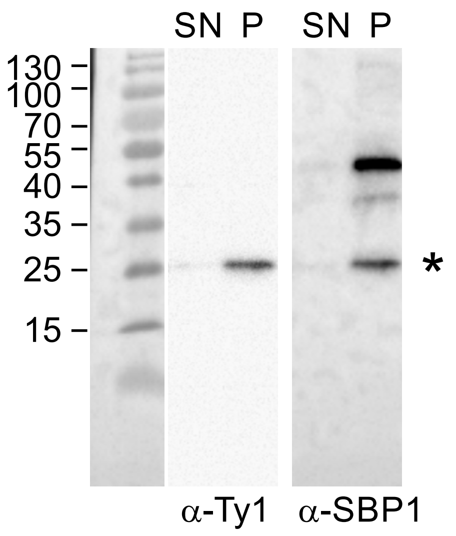

SN: supernatant

P: pellet

*: Leftover of Ty1 signal from first probing
Membrane was reprobed with α-SBP1

Figure 3C

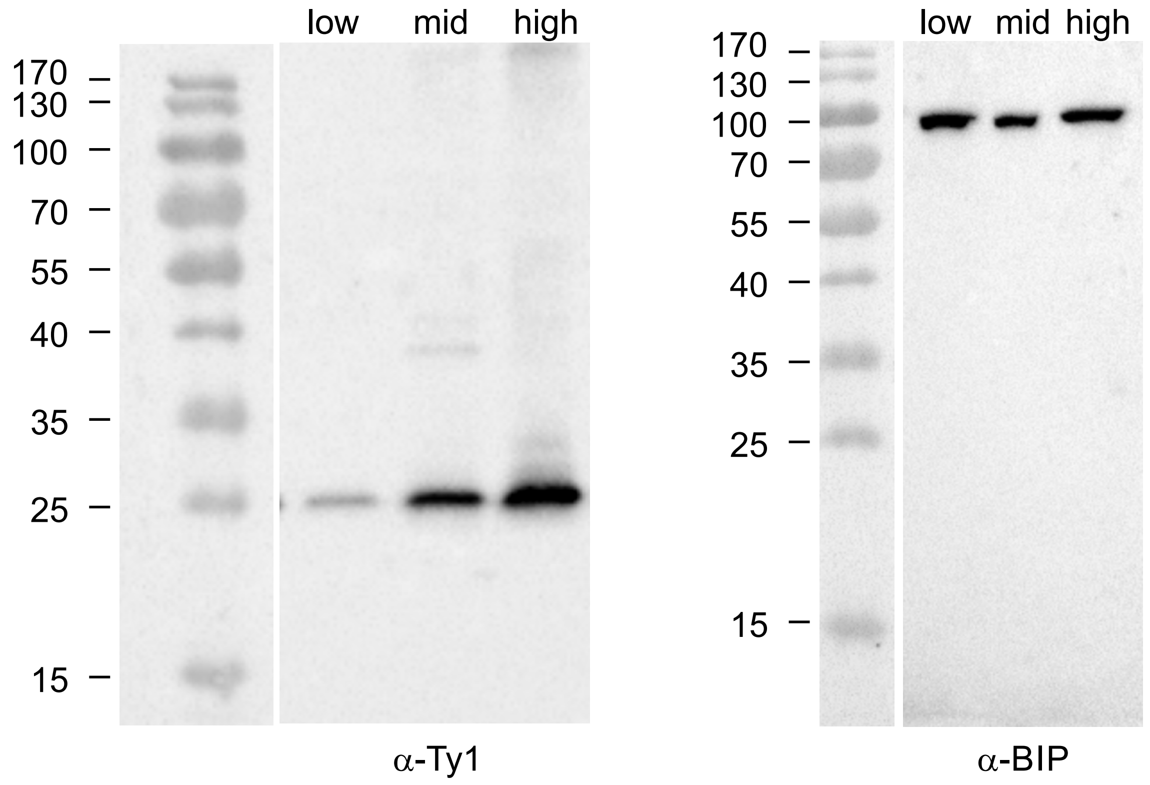

Figure 3E

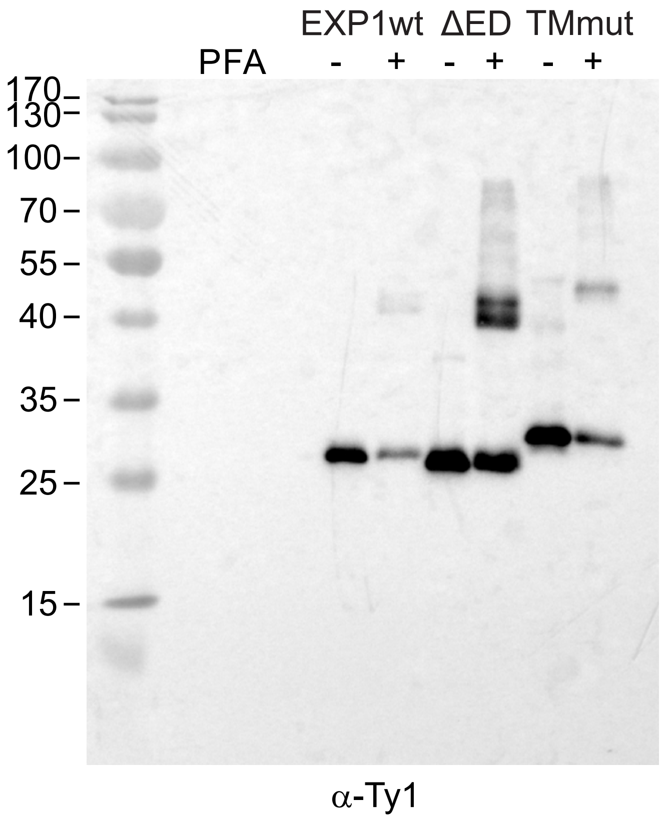

Figure 6I

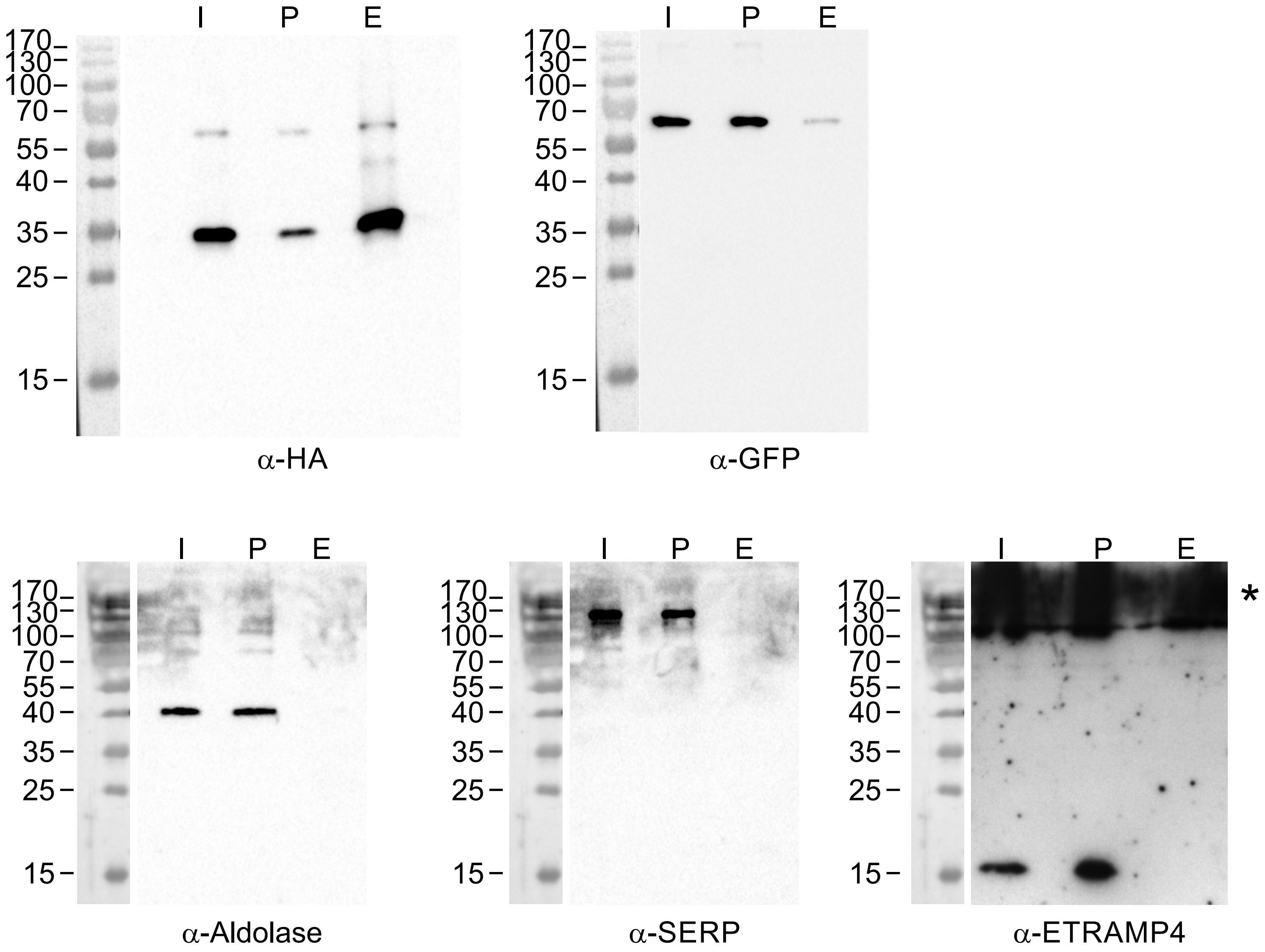

I: input , total lysate before IP
P: post-input, lysate after IP
E: eluate
*: unspecific background

S3B Fig

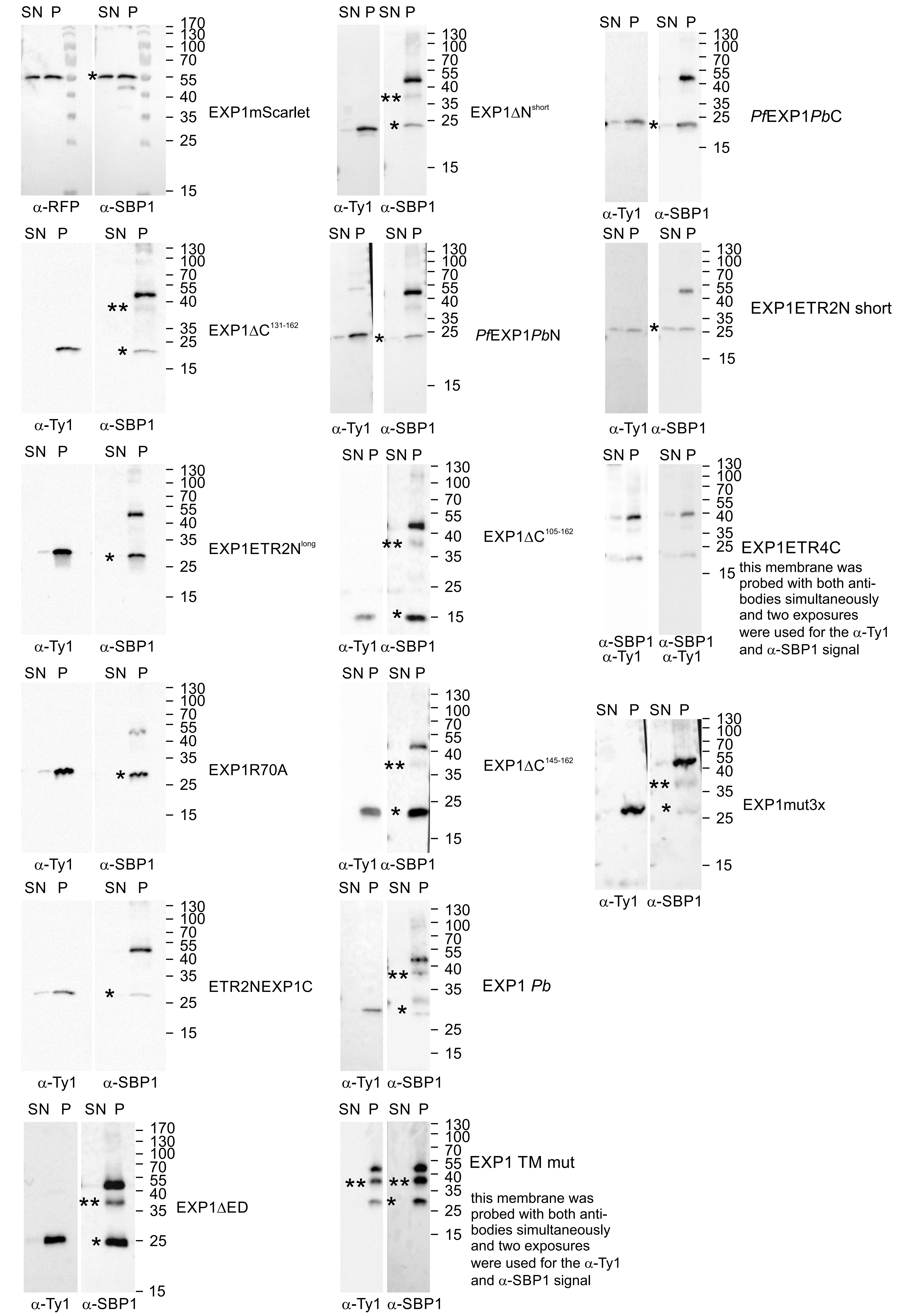

SN: supernatant P: pellet
All membranes were reprobed with α-SBP1 Leftover of Ty1 or RFP signal from first probing is indicated by an asterisk.
Double asterisk indicates SBP1 degradation band

S1I Fig

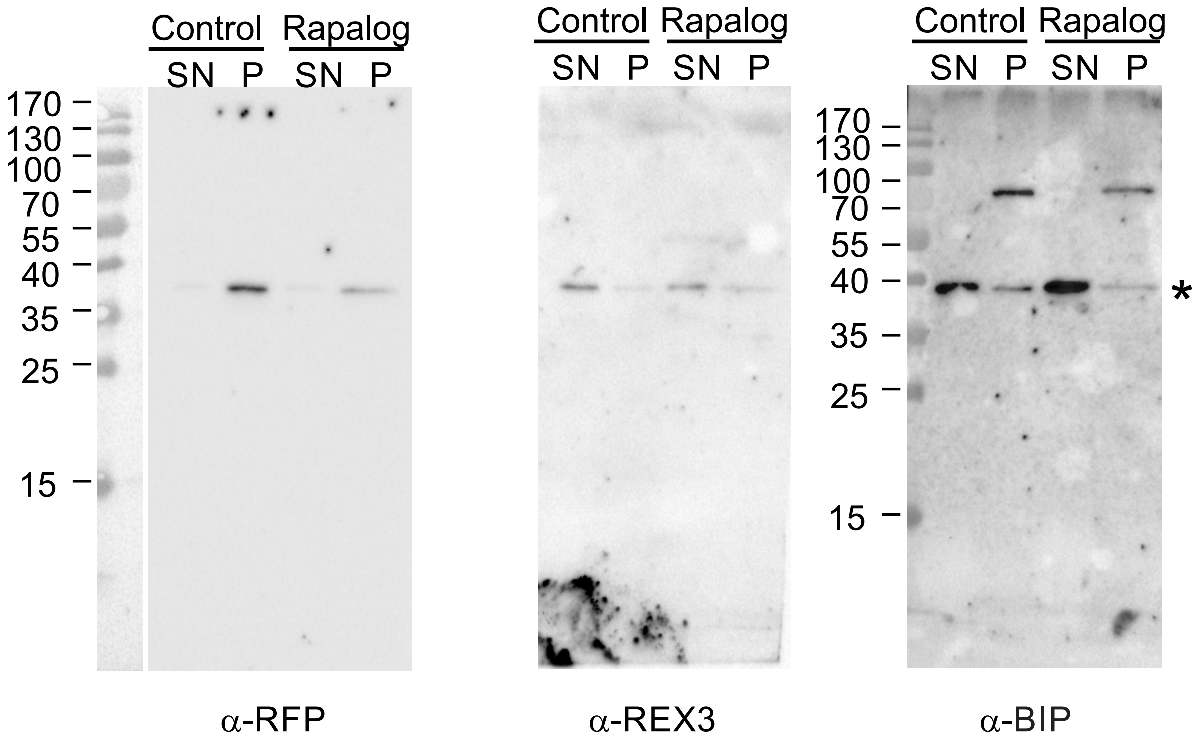

*: Leftover of REX3 signal from first probing
Membrane was reprobed with α-BIP

SN: supernatant

P: pellet

S6D Fig

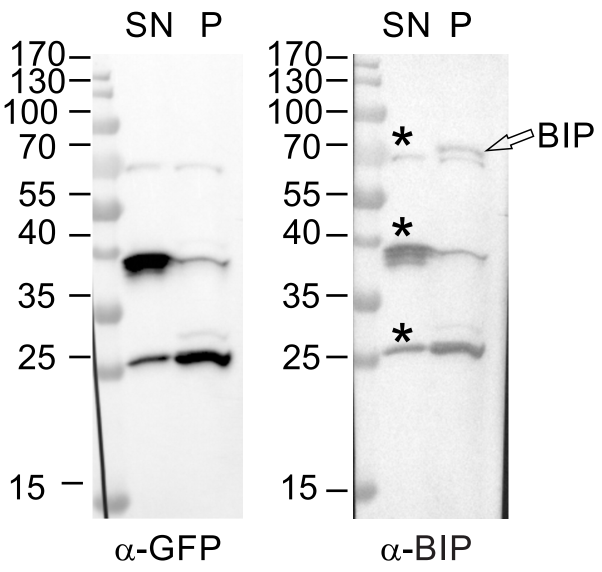

SN: supernatant

P: pellet

*: Leftover of GFP signal from first probing
Membrane was reprobed with α-BIP

S7C Fig upper panel

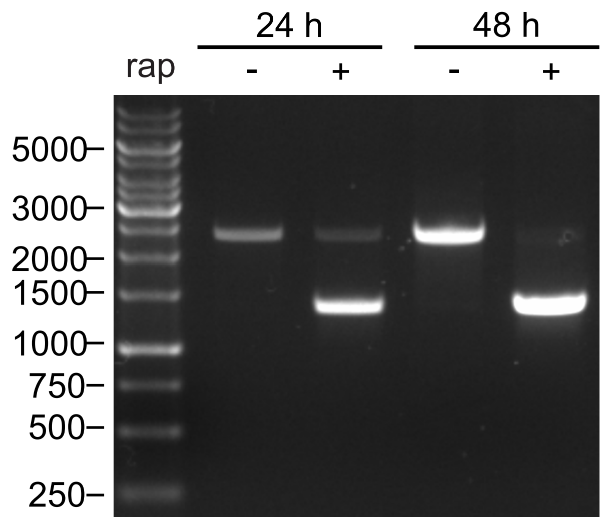

S7C Fig lower panel

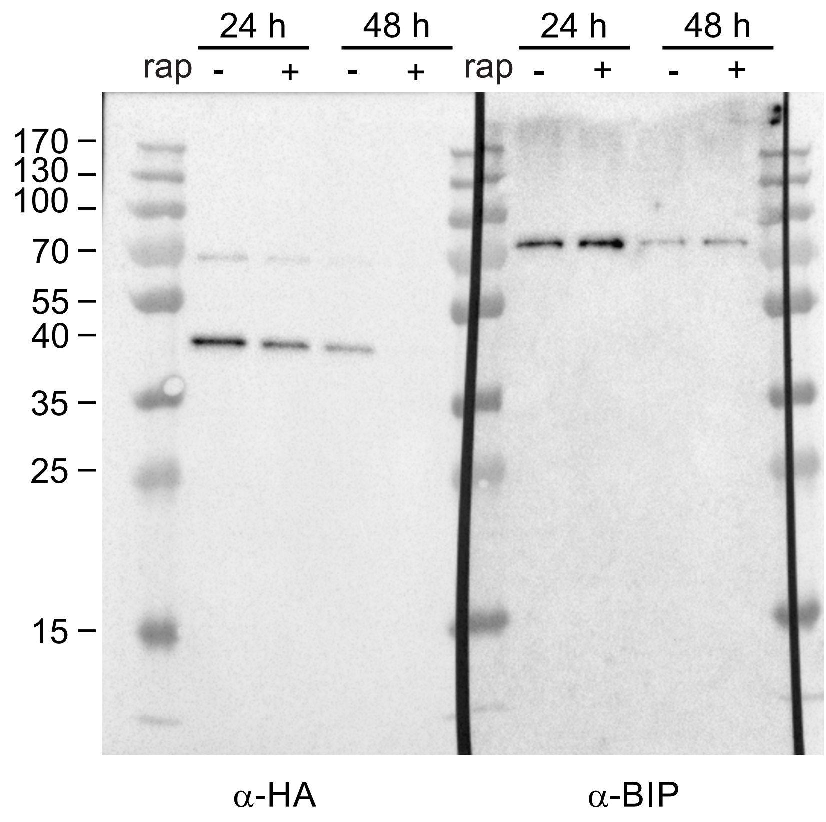

S8A Fig

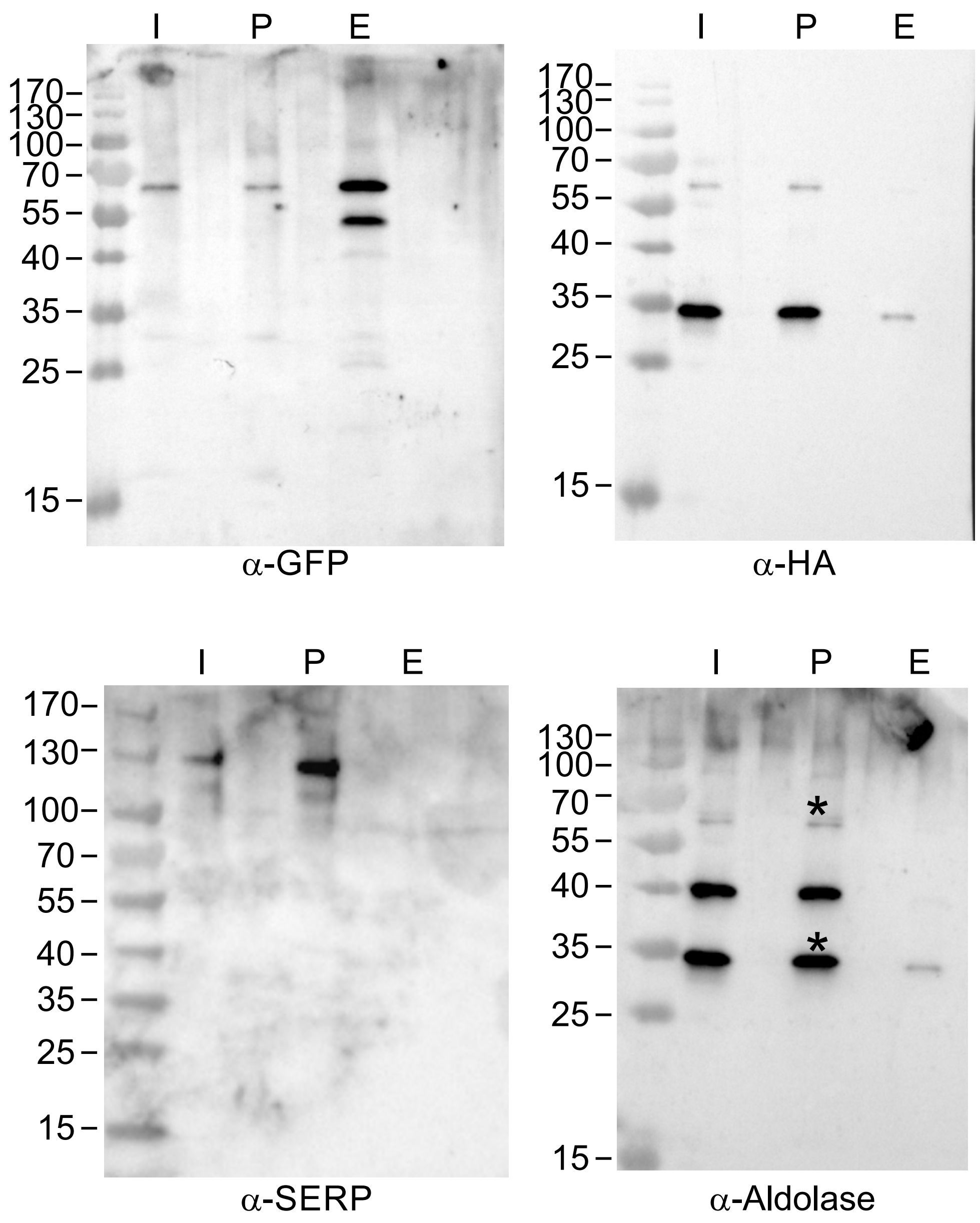

I: input , total lysate before IP
P: post-input, lysate after IP
E: eluate
*: Leftover of HA signal from first probing
Membrane was reprobed with α-aldolase

S8C Fig

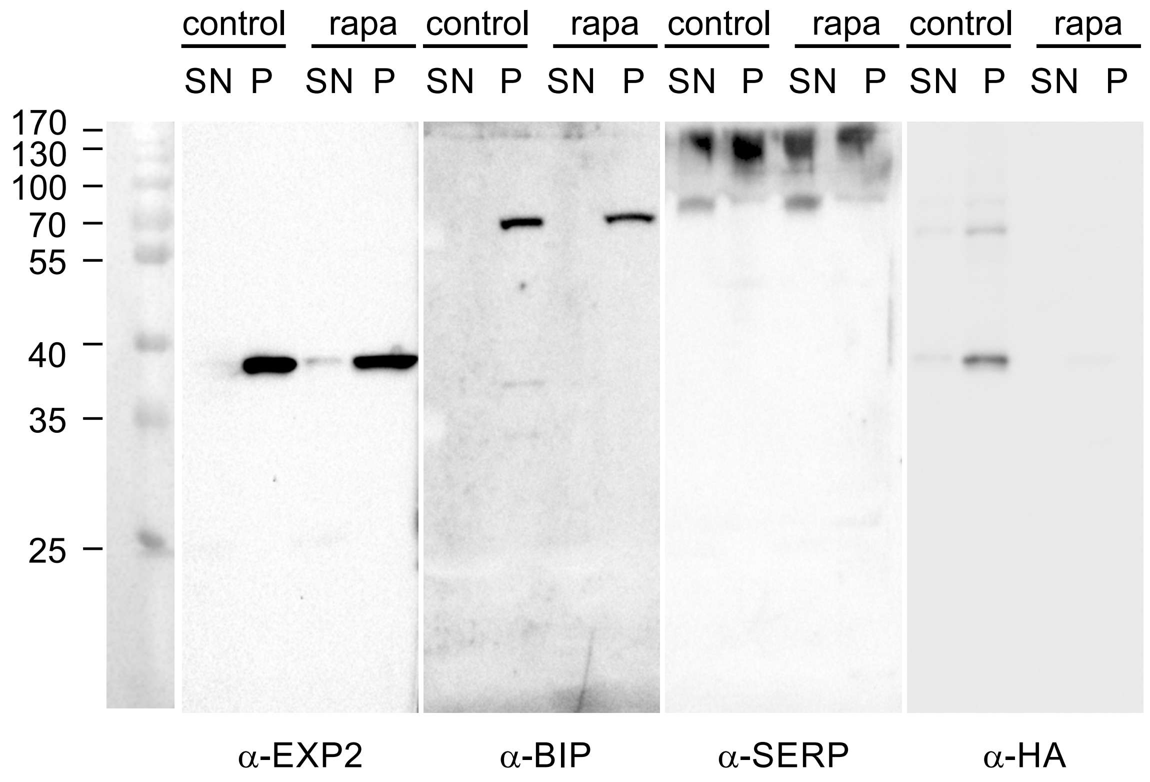

SN: supernatant

P: pellet

S8D Fig left panel

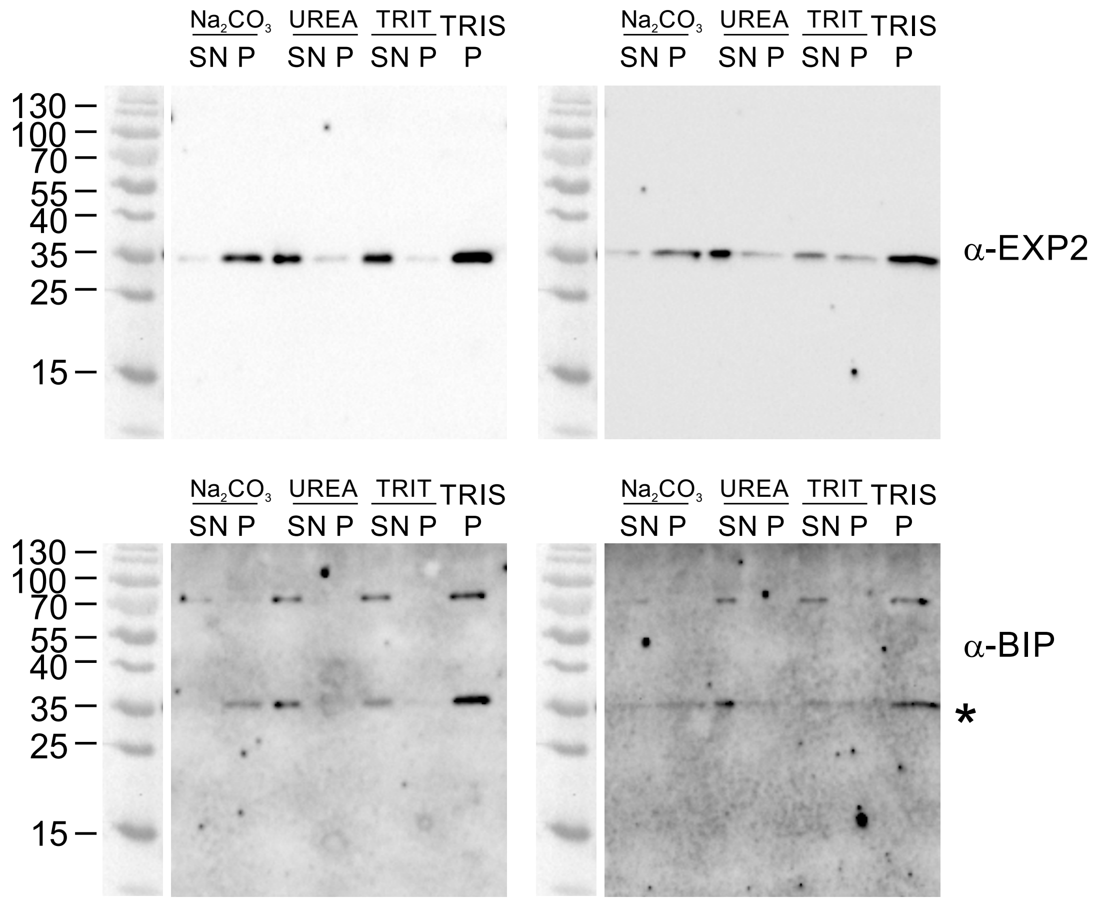

SN: supernatant

P: pellet

*: Leftover of EXP2 signal from first probing
Membrane was reprobed with α-BIP
