# Supplementary material for: EXP1 is critical for nutrient uptake across the parasitophorous vacuole membrane of malaria parasites
Source: PLoS Biol. 2019 Sep 30;17(9):e3000473. doi: 10.1371/journal.pbio.3000473 (PMC6786648; doi:10.1371/journal.pbio.3000473)
Supplement: Attachment — Submitted filename: Response to reviewers.pdf [file pbio.3000473.s013.pdf]

Editorial comments (our response in blue):

Thank you very much for submitting your manuscript "EXP1 is critical for nutrient uptake across the parasitophorous vacuole membrane of malaria parasites" for consideration as a Research Article at PLOS Biology. Your manuscript has been evaluated by the PLOS Biology editors, an Academic Editor with relevant expertise, and by several independent reviewers.

As you will read from the reviews (below), all the reviewers agree that the study is very well done. Reviewers #2 and #3 mainly request minor changes to improve the clarity and accessibility of the work. Reviewer #1 does suggest some additional experimental analyses. We have discussed the reviewers with the Academic Editor and feel there is really only one main outstanding question that needs some addressing which Reviewer 1 asks for experimentally and Reviewer 3 poses a question about - if EXP2 has two roles, one as a nutrient channel and the other for protein export (both of which require EXP2 to be inserted into the membrane) and EXP1 is required for EXP2 to correctly localise, why then is there a nutrient uptake defect but not a protein export defect when EXP1 is knocked down? How do you propose protein export EXP2 localises to the membrane without EXP1 and what is different about the two forms of EXP2? Reviewer 1 has asked for a solubility assay to determine if there is a change in the solubility profile of EXP2 after EXP1 knockdown, which we feel is not an arduous experiment to validate that EXP1 is indeed critical for EXP2 inserting into the membrane (which is only shown by IFA) and to explain these results. Reviewer 3 has asked for an explanation for the role of EXP1 in proper localisation of EXP2. We believe the experiment may certainly help to address/resolve this question and would encourage it.

We agree that this is an important point and carried out the solubility experiment initially using saponin, and then in more detail using carbonate and urea to extract peripheral membrane proteins. This revealed no significant difference in the solubility of EXP2 after loss of EXP1 (new S Fig 8c,d). We also added a model which summarises our data in the context of what is currently known. This model addresses points raised by reviewer 1 and 3 and explains how EXP1 can affect only the nutrient-permeable channel function of EXP2 but not PTEX (it indicates two complexes of EXP2 that are distinct in function, composition and expression timing, see details in response to reviewers).

Regarding the other experiments recommended by Reviewer 1 (Point 2 - pull downs with mutant forms of EXP1 to reveal which regions of this protein are important for interaction) - we acknowledge that these may be beyond the scope of this paper and will not consider it essential for publication. Additionally, Reviewer 2 asked for IFAs to analyse the localisation and fluorescent instensity of EXP1 during this time frame - once again, while welcome, we do not feel it is essential and will not insist on it for publication. Overall, we welcome resubmission of a revised manuscript that takes into account the reviewers' comments.

Due to the challenging nature of carrying out the suggested pull downs, this experiment was not done (the respective constructs poorly complement, making it difficult to obtain sufficient material for IPs, please see details in our response to point 2 of reviewer 1). We however now provide the IFAs to localise EXP1 during excision and quantified EXP1 levels based on Western blots (Wester blots are better suited for quantification of protein levels than IFAs, see response to the respective point of reviewer 2).

REVIEWS:

Reviewer #1:

Review of ‘EXP1 is critical for nutrient uptake across the parasitophorous vacuole membrane of malaria parasites’ by Mesén-Ramírez et al for PLoS Biology.

Overall Comments

This is a very interesting paper which incorporates an enormous amount of work and finally gives us insight into the crucial function of EXP1, an abundant Plasmodium protein. EXP1 was thought to be involved in resisting oxidative stress but probably has a more important function in helping intraerythrocytic parasite forms acquire nutrients across their enveloping vacuole membranes.

We very much thank the reviewer for this kind assessment.

Major Comments

1. In Fig 1 the authors establish using a very clever selection linked integration system that loxP mediated deletion of most of the exp1 gene arrests development of parasite blood stages. Detailed phenotyping of the mutants follows indicating they progress very slowly through the cell cycle after exp1 deletion. The young ring stage EXP1 null parasites produce more PVM protrusions than normal and often 'hug' the RBC surface. It was not clear to me if loss of exp1 is lethal for the parasite or do they continue to grow slowly? There is no real explanation of why the ring stage ΔEXP1 parasites appear abnormal and if this appearance can by reversed by complementation with the full-length wildtype EXP1. Is the deletion of the exp1 gene incomplete and do the wildtype parasites outgrow the exp1 mutants after a few cell cycles?

<u>Complementation of the ring stage phenotype:</u> We did not specifically assess the phenotype of the complementation in rings, as the complemented parasites grew almost at the same rate than the wild type in a 4 day growth assay (see Fig 2 and S 2 Fig). To confirm this finding, and to exclude that these parasites only show complementation of the trophozoite phenotype, we assessed the time of these parasites to complete the ring stage: the complemented parasites had a similar ring stage length to wt parasites whereas the ΔEXP1 parasites were severely delayed to reach the trophozoite stage. This experiment is now included as S 2d Fig. We did not carry out a long term time lapse analysis and detailed quantification of the ring stage phenotypes in the complemented parasites as the manuscript is already quite full and initial inspection of parasites did not indicate the presence of any of these phenotypes in the complemented parasites.

<u>Reasons for the ring stage phenotypes:</u> this is purely speculative, but we assume that this is also the result of limited nutrient supplies. At least the 'blebs' are visible in both, rings and trophozoites. We hope that the characterisation here might help comparisons with phenotypes of other mutants in the field which might further clarify the meaning.

<u>Slow progress or lethality of the EXP1 knock out:</u> Removal of EXP1 is lethal. The phenotype firstly leads to a severely delayed ring phase but many parasites still enter the trophozoite stage. However, these trophozoites do not complete the cycle and do not produce viable schizonts. This was stated in the text in line 116-119 of the manuscript (now line 122 in the track changes version of the manuscript): 'ΔEXP1 trophozoites did not complete schizogony...'. We realise that very slowly growing parasites (cycle time of > 4 days) would not be noticed, as they would not give rise to an increased parasitemia over the 4 day growth assay shown in Fig 1e. We therefore carried out a longer term growth experiment which would also test for the last part of this reviewer's comment (is loss of exp1 lethal or are there slow growers remaining?). It took 9 days before the ΔEXP1 parasites in the + rapalog sample showed a parasitemia high enough for analysis (5%) whereas controls had to be diluted multiple times (cumulative parasitemia of ~2000 %). PCR with the resurfaced parasites on rapalog on day 9 showed that they had an unexcised locus and only traces of the excised locus were detected. Hence, the parasites coming up after that time were breakthroughs expressing wtEXP1, not ΔEXP1 parasites. This further supports the conclusion that loss of EXP1 is lethal. These results are now included as S 1f Fig and described in the results (line 144ff in the track changes version of the manuscript).

2. In Fig 2 loss of EXP1* is complemented with wildtype EXP1 proteins under different strength promoters as well as with mutant forms of the EXP1 protein. This establishes that the complementing form of EXP1 needs to be strongly expressed and that residues in the transmembrane domain and ED region are important for function. While this section is informative as to what parts of EXP1 are important for parasite growth no information is provided as to why. Given that EXP1 appears critical for EXP2 nutrient pore function, pulldowns with the mutant forms of EXP1 might reveal which regions of this protein are important for interaction with EXP2.

We agree that it would be interesting to do pull downs with the mutated forms of the protein to assess if any of the domains are important for interaction with EXP2. However, we would like to point out that doing this is technically difficult as these mutations are lethal: either this would need to be done with the episomally overexpressed mutated EXP1 (which might not be meaningful as the endogenous functional copy is still present and might occupy functional space) or would need to be done in the parasites where the endogenous wt copy of *exp1* has been removed (the corresponding complemented ΔEXP1 parasites). However, due to the phenotype in these parasites and the failure to multiply, it would be challenging to obtain sufficient material for an IP. Please note that the editor indicated that this experiment is not absolutely necessary for the manuscript and due to the technical difficulty, we did not carry it out.

3. In Fig 3 the issue of whether or not EXP1 is important for protection of parasites from oxidative damage. This is because previous work reported that EXP1 was a glutathione S-transferase. The work presented here indicated that loss of EXP1 did not greatly sensitise the parasites to oxidative damage suggesting EXP1 possibly has another major function. The experiments are comprehensive and sufficiently cover the issue.

We thank the reviewer for this assessment.

4. In Fig 4 and S4 and it is shown the EXP1 null mutants can still export parasite proteins into the RBC compartment. Subsequent patch clamp analysis of the PVM indicates that current flow across the PVM is greatly reduced in the EXP1 null mutant. While I understand the basics of the Fig 4d what units are pA? Why does the rapalog scale show 0 pA and the control 35 pA? Was 0.2s the total measurement time or just the length of the scale bar beneath it?

We thank the reviewer for pointing out that understanding the figure would be easier with more explanation. We did this by changing the legend to read (changes in red):

"Left, current recorded from liberated control and ΔEXP1 parasites (rapalog) at 30 mV applied potential to the pipette electrode. Scale bar shows time in seconds and current in Picoampere (pA). The dotted line indicates a current reference level. At 30 mV the PVM channels have an open probably of about ½, hence the flicker is offset in the control example as multiple channels are in the recording. The shown recordings are representative of the experiments done in each condition and show 1s details from longer recordings to resolve the typical channel flicker in print. Right, probability of detecting at least one PVM channel per sealed patch (fchan) in ΔEXP1 parasites (rapalog, N=14) and controls (N=12). Fischer's exact test was used to estimate P value. Error bars indicate standard deviation."

5. In Fig 5 the localisation of EXP2 is examined in EXP1 null parasites and found to be aberrant being concentrated into PVM protrusions and bodies around the parasite. ETRAMP5 was similarly affected but not other ETRAMPs. The implication if this finding is that EXP2 requires EXP1 for an even localisation around the PVM and pulldown experiments indicate the two proteins interact. The result that EXP1 is required for EXP2 function is a curious finding for it would be expected to reduce both

nutrient uptake as well as protein export since EXP2 forms both nutrient pores and the protein translocon pore of PTEX. Up until this EXP1 null result I would have presumed the heptameric EXP2 pore structure would be similar in both nutrient and translocon complexes but the EXP1 result suggests the nutrient pore is a distinct and possibly different structure. This could be explored by determining if EXP2 can form a membrane pore in EXP1 null parasites by determining if EXP2 is still membrane associated when EXP1 is absent (ie, does EXP2 become soluble without EXP1?). Also, does the EXP2 protein that is pulled down with EXP1 lack any association with the rest of PTEX?

This is a very interesting point on this finding that we have found very intriguing. It indeed suggests that there are 2 pools of EXP2-containing complexes that are of different composition and serve different functions. This fits well with recent data that shows that EXP2 is present in two pools, an early expressed one associated with the other PTEX components and a second, later expressed pool that is devoid of the PTEX component HSP101 (Garten et al., 2018). Garten et al 2018 and earlier work on PTEX (Bullen et al 2012) also noted that EXP2 has a different expression profile compared to the other PTEX components: EXP2 peaks mid cycle, whereas the other PTEX members peak in ring stages. Interestingly, EXP1 has an expression profile matching EXP2 but not other PTEX components (this is now highlighted in Fig 8). In taking with this expression pattern, our findings indicate that EXP1 is needed specifically for the function of this second, PTEX-free, EXP2 pool. This explains why loss of EXP1 affects the location of EXP2 only in trophozoites and specifically affects the nutrient permeable channel function but not protein export. Therefore, EXP1 permits one to specifically study the activity of this second pool of EXP2. Overall this indicates that in rings, when protein export is a predominant requirement for the parasite, EXP2 exists predominantly in PTEX. In trophozoites, when protein export is less important but nutrient acquisition for rapid growth is critical, the major EXP2 pool is needed for nutrient uptake. In response to this point and to reviewer 3 (see below) we now provide a model (new Fig 8) that summarises our data in this context and indicates two different EXP2 complexes that are distinct in function, composition and expression timing. In addition, discussion of this topic was expanded (line 386ff and line 398ff in the track changes version of the manuscript).

We also carried out the solubility experiment suggested by the reviewer to check if EXP2 became soluble in the EXP1 KO. This experiment showed that EXP2 was still membrane associated after EXP1 had been deleted (New S8c Fig). Next, we tested in more detail if the membrane association properties of EXP2 changes when EXP1 is missing by using carbonate and urea extractions. However, no significant alteration in the membrane-extractability of EXP2 was detectable in the EXP1 KO compared control (New S8d Fig). It is therefore likely that the functional inactivation of EXP2 after removal of EXP1 is not due to a changed insertion into the PVM.

6. In Fig 6 the growth of parasites was examined on nutrient limited media to show that this sensitised parasites to grow slowly particularly when EXP1 was deleted or expressed at a low level. Reduction of EXP1 expression appears to reduce the capacity of nutrients across the PVM particularly when nutrients are in limited supply. The minimal media appears to have reduced levels of amino acids but which amino acids? Only isoleucine is essential with the remaining amino acids being produced from haemoglobin breakdown. Was this experiment repeated with only isoleucine being limited? Part C should have p values between important data pairs. In part E which columns are treated with azide versus no azide and which are minimal versus complete medium.

Initial tests showed that medium lacking all amino acids but isoleucine (382 and 200 μM isoleucine) did not support parasite growth. We therefore used the medium lacking amino acids and added dilutions of the standard medium, thereby obtaining medium with low concentrations of amino acids. Testing growth of 3D7 in these test mediums showed that adding 1/20 of standard medium (resulting in an isoleucine concentration of 20 μM, which should support parasite growth according to Babbit et al., 2012) permitted limited parasite growth (see graph below):

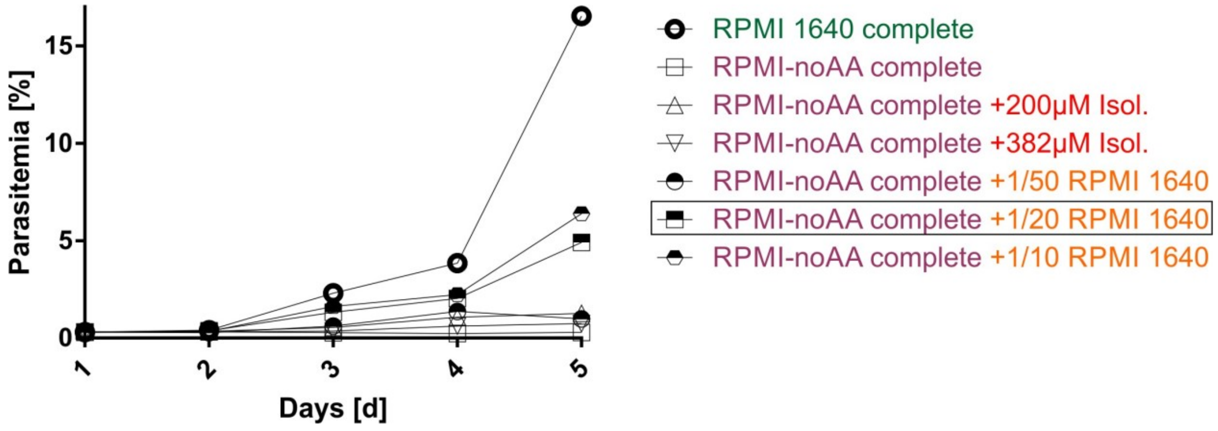

We therefore used this medium, containing 1/20 of the amino acids in standard medium, for our assays. These tests also indicated that beside isoleucine, small amounts of other amino acids were needed to permit parasite growth (or that parasites would first need to adapt to growing with isoleucine alone). Preparation of this medium is now more accurately explained in the materials and methods.

We now included P values for important data pairs in Fig 6c.

We apologise for not better explaining Fig 6e (now Fig 7e). We now included a more detailed explanation what is shown in this graph into the figure legend which now reads: '**(e),** Growth on day 4 (2 cycles) of the indicated parasite lines and condition (± rapalog) in presence of azide in proportion to growth of the same parasites in medium without azide (left, NaN_3 vs no NaN_3) or growth in amino acid-limited medium in proportion to the same parasites grown in complete medium (right, limiting vs complete medium). Growth of the control culture (medium without NaN3 or complete medium) was set as 100%. Rapalog (rapa) was added one cycle prior to the growth test to start with the corresponding knock out parasites. Green line indicates mean of at least *N*=5 independent experiments.'

Minor Comments.

7. Fig 1. Part K right. What are the band sizes of the Kb ladder? Maybe label as per part C. In part L right, what do the red boxes mean? Is '2A' in the gene diagram the same as T2A in the legend?

The ladder in Fig 1k (now Fig 2f) was included. The red boxes in what is now Fig 2g were replaced with a scatter plot showing individual values and the mean and SD was added. The legend now refers to '2A' as the T2A skip peptide.

8. Indicate cell cycle number in Fig 1d and e.

This is now indicated.

9. Line 439 is 'Light blue arrow heads show blebs.' in part E mean to be in part F?

Both, part (e) and (f) have light blue arrows showing blebs (in part (e) this is visible in the example giemsa smears shown beneath the graph).

10. Line 450 What gene is Lyn-Cherry?

We introduced Lyn-mCherry in line 133 (now line 143 in the track changes version of the manuscript) and added a reference (Birnbaum et al., 2017, first use in P. falciparum) and (Inoue et al., 2005, first description). The construct contains the plasma membrane targeting signal of the Lyn kinase (MGCIKSKGKDSAGA), a commonly used construct in other systems. Generation of the mCherry fusion construct is described in the cited paper (Birnbaum et al., 2017).

11. In Fig 2A size bars are missing from the IFA panel.

This was corrected.

12. What do the coloured boxes in 2D mean?

We now removed the boxes and provide a scatter plot showing the individual data points, mean and SD.

13. Line 569. I could not find Table S1.

We apologise for this, Table S1 is now included.
We thank the reviewer for pointing out these errors.

--
Reviewer #2 (signs review as Markus Meissner):
Summary
In this study the Spielmann group performs an extensive functional characterisation of PfEXP1. This protein has been described previously as essential for the asexual development of Plasmodium spp and multiple, potential roles, including involvement in artemisinin resistance, resistance to oxidative stress or interactions and uptake of host apolipoprotein have been suggested by others.
In this study, a conditional mutant for Exp1 was generated using the DiCre system, which allowed the authors to perform a very thorough and detailed analysis of the resulting phenotype, using a combination of very neat, cutting edge technologies.
Briefly the authors convincingly demonstrated that:

- Exp1 is essential and parasites fail to replicate in absence of Exp1
- A carefully performed complementation analysis demonstrates that full length Exp1 is required for its essential function. Interestingly, the analysis indicates that there is only poor functional conservation within apicomplexans, since Exp1 of P.berghei appears to complement poorly for PfExp1.

Furthermore, the previous hypothesis that EXP1 is crucial for protection against oxidative stress has been convincingly invalidated, since mutations in the GST-domain did not result in loss of complementation. Similarly, the previous suggestion that EXP1 is involved in artemisinin resistance was invalidated.

- Loss of GST activity results only in minor effects on parasite growth and EXP1-activity must be crucial for other functions.
- Using patch Clamp measurements, the authors convincingly show that Exp1 is essential for the function of the major nutrient permeable channel of P.falciparum, a major and important finding!
- EXP1 is required for “smooth” localisation of EXP2, but not for PTEX function itself
- EXP1 and EXP2 can interact
- EXP1 is required for nutrient uptake, but not PSAC activity at the RBC membrane

Own opinion:
This is a very careful and exhaustive analysis that should be the gold-standard for a proper characterisation of phenotypes. It leaves very little room for major criticism, since the authors took

great care to perform many control experiments, so that the final conclusions are fully supported by the data. In fact, the authors provide so many data and controls that it would have been sufficient for a separate paper.
Most importantly, this study can be seen as a milestone in order to understand the dual function(s) and differential regulations of Exp1 and Exp2 in protein export and nutrient acquisition. It will also end speculations regarding additional functions/roles of Exp1 for drug resistance or resistance to oxidative stress.
There is no doubt that it will be of high interest to a broad readership.
My comments are relatively minor, since I didn't find any data/conclusions that require additional experimental validation.
We very much thank the reviewer for this kind assessment.

Minor comments:

- The floxed locus is myc- and HA-tagged. Did the author also use alpha-myc to check for expression of truncated construct?

We did check for the truncated construct but this stub is detectable only by IFA, not Western blot, likely due to its small size and because its amounts seems be quite low (probably this small fragment is rapidly degraded). An IFA is now included in S 1d Fig and this is mentioned in the main text (lines 115/116 in the track changes version of the manuscript). Please note that after excision, the stump also receives the HA tag (S 1a Fig), hence the HA signal overlapping with the stump in the rapalog sample of the second cycle in S 1d Fig.

- Fig.1c: The authors present WB and PCR of cultures induced with Rap. It seems there is no significant downregulation within the first 24h and Exp1 is fully lost during the second cycle. It would be helpful if the authors could also provide IFAs to analyse the localisation and fluorescent intensity of Exp1 during this time frame.

Quantification of the knock out efficiency is a good point. We added an IFA for the 24 h time point (S 1d Fig, see previous point) but please note that anti-HA will also pick up the truncated fragment after excision occurred (see previous point). This precludes an IFA-based quantification. In addition, quantifying IFA signals is somewhat treacherous. We therefore carried out densitometry for the Western blot to track loss of EXP1 (n = 2). This indicated a reduction of EXP1 to 72,9 % (+/- 3,4 %) in the first cycle and down to ~4,7% (+/- 2,1 %) at the 48 h time point (2nd cycle). The quantification of the Western blot was added to Fig 1 (new panel d) and this is now mentioned in the text (lines 108ff in the track changes version of the manuscript).

- The description of the figures in the text is sometimes confusing. For example in line 132 its mentioned: (Fig.1f, arrows). There are 4 differently coloured arrows in Fig.1f. Other examples throughout the manuscript. This should be thoroughly corrected in the revision.

We thank the reviewer for pointing this out, we specified the arrows mentioned in the text where necessary.

- It is recommended that the authors describe some details of their patch-clamp method presented in figure 4 already in the result session for better understanding and flow of the manuscript.

We thank the reviewer for this advice. We changed the paragraph to read:
"To test if EXP1 affects the nutrient-permeable channel activity at the PVM, patch-clamp measurements were performed on the PVM of ΔEXP1 parasites liberated from their host cell. After liberation, the PVM remained intact, as evidenced by the retention of a co-expressed soluble PV

marker (Fig. 4c and Supplementary Fig. 1g). The PVM of the liberated parasites was now accessible to a patch-clamp pipette. After giga-seal formation each individual sample was inspected for channel activity, defined as a current flicker from closing channels at 30 mV applied voltage to the patch pipette (Garten et al. 2018). While channel activity was often immediately apparent in the control sample, channel activity was absent from most of the ΔEXP1 parasites (Fig. 4d left). The frequency to detect at least one channel at the PVM (fchan) of ΔEXP1 parasites was significantly reduced compared to controls (Fig. 4d right). Together these results, demonstrate that EXP1 is important for the nutrient-permeable channel activity at the PVM but not for protein export."

- Figure 5 is slightly overloaded. The IFAs using antibodies for EXP2 (5d,e) can easily be moved to supplementary figures, since it shows the same as 5a,b

We realise that the figure is quite crowded but also think that showing the effect on the endogenous protein is quite important. We would therefore prefer to retain this panel in the main figure. We however rearranged the figure to make it appear less crowded.

- In the discussion the authors should also consider recent results from other apicomplexan parasites, such as Gold et al., 2015, which are nicely supporting the data presented in this study.

This work was cited in the introduction (lines 63-65) and the discussion (line 354, together with the corresponding data in Pf). We now more explicitly mention this work when discussing the model that EXP2 likely is part of two distinct complexes (lines 386ff in the track changes version of the manuscript).

--

Reviewer #3:

Review for submission of manuscript PBIOLOGY-D-19-01632R1

By Paolo Mesén-Ramírez, Bärbel Bergmann, Thuy Tuyen Tran, Matthias Garten, Jan Stäcker, Isabel Naranjo-Prado, Katharina Höhn, Joshua Zimmerberg, Tobias Spielmann

"EXP1 is critical for nutrient uptake across the parasitophorous vacuole membrane of malaria parasites"

Mesén-Ramírez et al. report their findings concerning the parasitophorous vacuolar membrane protein Exported protein-1 in the apicomplexan parasite P. falciparum causing malaria. They characterize its properties as a nutrient conducting trans-membrane channel and also its functional and physical association with Exported protein-2, another vacuolar membrane protein that functions as a protein-conducting channel in the vacuolar translocon PTEX but also serves as a nutrient-conducting transmembrane channel.
Because nutrient uptake is essential to parasite survival and its expansion inside the infected red blood cell, there is considerable interest in "channeling the vacuole" by characterizing these nutrient or protein "channels" to better understand parasite physiology. This type of studies potentially extends the number of drug-targets by identifying novel essential pathways, the so-called permeability pathways, and their molecular effectors to design much-needed new anti-malarial compounds.

The work shows that EXP1 is needed for nutrient uptake at the vacuolar membrane, required for proper vacuolar localization of the pore-forming protein conducting channel EXP2, a core subunit of EXP2 but also a nutrient permeable channel as demonstrated by Garten et al. in a previous study.

I think this is beautiful work in terms of cellular and molecular apicomplexan parasitology that significantly expands the current knowledge of the field. I have a few questions and objections that the Authors may want to address but I strongly support publication once these issues have been addressed.
We very much thank the reviewer for this kind assessment.

Comments and questions.
Line 37. What do authors means by "major" in major integral PVM protein EXP1? It is unclear to me. Is it abundance?
We realise this wording is ambiguous and thank the reviewer for pointing this out. We replaced 'major' with 'abundant'.

Line 41. I would suggest saying 'pore-forming protein' (rather than molecule)
This was changed as suggested.

Line 61. I would suggest being more precise "which forms a heptameric PVM-spanning channel". It does not hurt.
This was changed as suggested.

Authors use conditional KO of the gene exp1 to show that the protein EXP1 is essential for propagation in RBCs. Ring stages are slow to reach the trophozoite stages and schizogony is interrupted. Thus the effects are quite drastic and remarkable. The pervasive effects of EXP1 KO even affect the gametocytes but to a lower extent. The cytology indicates the presence of what Authors call 'blebs' protruding out of the PVM although the integrity of the PVM is not compromised it is obviously altered at the ultra-structural level. The methodology and analysis are extremely thorough and beautifully well illustrated in the figures.

Although I find the section describing the complementation (or lack of) of the cornucopia of chimeric EXP1 constructs tested by the Authors to probe the role of the different regions in protein functionality quite complex, it is remarkable work. I am glad the Authors included Supp Fig 2 otherwise it would be a nightmare to understand it.
We thank the reviewer for the positive assessment of these points.

Paragraph starting line 167.
Based on high-resolution crystallographic structures of membrane proteins (MP), GXXG motifs are known to be essential for very close TM helix to TM helix packing/contact interactions in MP structures (whether it is intramolecularly as shown in Aquaporins for example) or between molecules to favor tightly packed oligomers inside the bilayer. Unless I missed where it is described, what kind of point mutations did the Authors introduce in the two GXXG motifs.
We apologise that this is not immediately obvious, it is listed in S 2a Fig in the schematics. We now added this information to the main text. The Gs were changed to L, a common transmembrane alpha-helix residue.

Line 161-163. "EXP1 lacking a 10 amino acid stretch in the N-terminus named E-domain (EXP1ΔED), a region proposed to be necessary for the dimerization based on similarity with MAPEG complemented only poorly (Fig. 2b). However, EXP1ΔED was still capable to homo-dimerize (Fig. 2e), indicating that the loss of function was not related to the capacity to oligomerize."

Fig 2e. What is the sequence of the so-called E ‘domain’ or better said 10-aminoacid long protein segment?

The sequence is SGVSSKKKNKK and this is now indicated in the main text (lines 175/176 in the track changes version of the manuscript). We also noted that the region previously speculated to correspond to the E domain is in fact 11 amino acid and this is what we deleted in this construct. This has now been amended in the text.

Why would ΔED deletions then seem to form more dimer as suggested on Fig. 2e ... if that band actually meaningful? As a membrane protein biochemist, It do not really know what to do with what the Authors show on Fig2e concerning dimerization of their constructs wtEXP1 compared to the TMmut and ΔED variants. Honestly, I am very skeptical about this. MPs display erratic electrophoretic behaviors on (denaturing) gels as a function of the detergent environment and fraction preparation (among other things). I am not convinced the Fig and results substantiate the claims made by the Authors when it comes to association state.

We established in previous work that EXP1 forms homo-oligomers. The oligomers become detectable after formaldehyde crosslinking of intact parasites (Spielmann et al., 2006 MolMicrobiol). This for instance also included experiments that showed that if a non-membrane permeable crosslinker (BS3) was used, the oligomers were only detected if this crosslinker was permitted access to the PVM, demonstrating that the oligomers were not due to other sample conditions but depended on the actual crosslinking at the PVM (Spielmann et al., 2006). Based on our previous results we are therefore confident that EXP1 forms oligomers. The bands detected in the Western blot with extracts of formaldehyde crosslinked parasites in Fig 2e correspond to these previously detected oligomers. We agree with the reviewer that this should not be over-interpreted as it is a rather crude analysis. However, we would also like to stress that this data only shows that there is no gross change in oligomerisation. This is useful information, as it could be assumed that if EXP1 indeed is a MAPEG and the E domain serves the proposed function, oligomerisation could have been ablated. We now further cautioned this point in the text which now reads: 'Previous work indicated that EXP1 homo-oligomerises (Spielmann et al 2006). However, loss of function of EXP1ΔED was not due to profound alterations in its capacity to oligomerize, as dimers were still detectable after formaldehyde crosslinking (Fig 2e).'

Based on this comment, this makes the statement on lines 172-173 a bit dubious in my opinion. Based on their extensive complementation assays I agree that all regions (rather than domains, the only domain is the TMD after all) are important to protein function(s) but I would not conclude any further.

We agree and removed the statement about the membrane domain. We also exchanged 'domains' in the title and body of this section with 'regions'.

The sections on the role of EXP1 in coping with oxidative stress or its effect on artemisin resistance are clear and the results well presented.

The GST related section is clear. I must say that as a biochemist and structural biologist that read the article in Cell (reference 16 from Lisewski et al), it makes me want to cry when I see how poorly characterized the so-called recombinant and pure EXP1 protein expressed in E. coli was in this study. One wonders if it is at all a GST…

We fully agree that it is unclear if EXP1 is a GST at all. However, we can’t formally exclude it; we can only conclude that the GST function is not essential.

Since the previously postulated function as a heme-detoxifying GST did not appear to be responsible for the phenotype in ΔEXP1 parasites, the Authors look at other possible pathways and possibly the interaction between EXP1 and EXP2, the PTEX subunit, and thus protein vacuolar trafficking.

If memory serves, EXP1 was not initially identified as a main interacting partner with PTEX (via EXP2 it seems based on this study) in the initial proteomic-based approaches (most from de Koning-Ward's group). While subsequent work by this group (this work and reference 30 in 2016) shows IPs with interaction between EXP1 and EXP2. Would the Authors offer an explanation about this?
This is a very interesting point. The explanation likely lies in the fact that there are two EXP2 complexes of different composition: PTEX, which contains EXP2-PTEX150-HSP101 and the nutrient permeable channels which contains EXP2 and associates with EXP1. Only pull downs with EXP2 would therefore purify both complexes. To our knowledge the original PTEX work pulled down the complex using PTEX150 and HSP101, hence ending up with PTEX only (de Koning Ward et al., 2009; Bullen et al., 2012). When EXP2 was used for IPs in these studies, this was only done to confirm the presence of the other PTEX components using specific antibodies in Western blots but no proteome was generated. Later studies carried out mass spectrometry of material pulled down using EXP2 antibodies, but the full data was not shown. Rather the enrichment of specific components was assessed (e.g. Elsworth et al., 2016) and it is therefore not clear if EXP1 was detected at all or enriched. In Mesen-Ramirez et al., 2016, we carried out mass spectrometry with the material pulled down with EXP2 (using anti-HA and a parasite line with endogenously HA-tagged EXP2) and obtained the components of both EXP2 complexes, including EXP1. The PTEX pull downs also frequently used ring stages which contain a high proportion of PTEX associated EXP2 but the amount of nutrient permeable channel is likely low (see also new Fig 8).

Overall the published pull down data therefore supports the model that there are two pools of EXP2 that differ in composition and function. This agrees with the previous experimental finding that there is a late EXP2 pool devoid of PTEX components (Garten et al 2018), a possibility speculated already before (Gold et al 2015) and together with our data gives rise to the model now added as new Fig 8 (see also discussion on this topic in response to reviewer 1 and for the model below).

Line 249. Typo
"ΔEXP1 parasites showed NO (instead of not) defect in the export of 249 SBP1, REX1, REX2 …"
This was corrected.

Line 307.
"Parasite surface ANION (instead of anyone) channel"
This was corrected.

"Loss of EXP1 abolished the correct localisation of EXP2, a pore-forming molecule required for the nutrient-permeable channel activity and protein export at the PVM. Unexpectedly loss of EXP1 however affected only the nutrient-permeable channel activity of the PVM but not protein export."
Could they offer an explanation for the role of EXP1 in proper localization of EXP2?
We now discuss 2 possibilities how EXP1 could affect EXP2 (line 420ff in the track changes version of the manuscript) and included them into the model added in response to the points below (indirect role: e.g. EXP1 prevents aggregation of EXP2 pores; direct role: EXP1 is a part of the nutrient-permeable channel itself and its absence leads to an incomplete, defective channel).

The Authors show that EXP1 controls the localization of EXP2 but does not affect protein trafficking mediated by EXP2 through PTEX and that only the NP channel activity is defective in ΔEXP1 parasites: that activity is vacuolar not at the iRBC membrane surface. They also show that the two proteins interact in reciprocal IPs. EXP1 depleted cells are susceptible to amino-acid starvation.

All their results are clear and support their conclusions. These are delicate experiments combining powerful genetic tools in Plasmodium and patch-clamp based measurements on parasites released from their host RB cell.
They demonstrate that EXP1 is required for EXP2-based NP channel activity and functionally distinguish EXP2 roles in nutrient uptake and protein export. Does this hint again at two molecular pools of EXP2: one associated with EXP1 for nutrient uptake and one associated with the rest of the PTEX core components (PTEX150, HSP101) and other ancillary proteins?
Yes, two molecular pools is the most straight forward explanation. This interpretation of our data fits very well with the discovery of a large pool of EXP2 in trophozoites that is devoid of the PTEX component HSP101 (Garten et al., 2018) and we believe our EXP1 knock out functionally impacts only this nutrient-permeable channel specific pool. This is now better discussed (line 386ff and 389ff in the track changes version of the manuscript) and included a model (new Fig 8, see also next point and response to reviewer 1).

Thus, could the Authors make a simple figure that summarizes their model on how EXP1 (and EXP2) interplay in the cell.
This is an excellent suggestion; this is now included as new Fig 8. The model is not simple, but we hope it adequately summarises our data in the context of what is known and makes it more accessible to a general readership.

They hint at their model at lines 361-362. "Thus, the expression of two functions, nutrient uptake and protein export, are at least differentially regulated and at most molecularly distinct."
It is a model and is likely to be imperfect but at least it would summarize their findings in a simple way more easily understandable by a non-specialist reader.
We agree that the discussion was kept too short to sufficiently explain this. We now expanded the discussion on this topic (line 386ff and 389ff in the track changes version of the manuscript) and provide a model (see previous point).
